# Chemical structure drives developmental toxicity of alkyl-substituted naphthalenes in zebrafish

**DOI:** 10.1016/j.envint.2025.109837

**Published:** 2025-10-01

**Authors:** Mackenzie L. Morshead, Lisa Truong, Steven J. Carrell, Richard Scott, Kim A. Anderson, Robyn L. Tanguay

**Affiliations:** Sinnhuber aquatic Research Center, Department of Environmental and Molecular Toxicology and the Oregon State University Superfund Center, Oregon State University, ALS 1007, Corvallis, OR 97331, USA

**Keywords:** Polycyclic aromatic hydrocarbons, Naphthalene, Alkyl-substituted naphthalenes, Zebrafish, Transcriptomics, Molecular docking, Narcosis

## Abstract

Naphthalene and its alkyl-substituted derivatives are among the most abundant polycyclic aromatic hydrocarbons (PAHs) in environmental and human exposure studies, yet their developmental toxicity and mode of action remain poorly understood due to challenges in testing semi-volatile compounds. This study developed a vial based, high throughput method to effectively assess the activity of naphthalenes and a set of 24 alkyl-substituted naphthalenes. Early life stage zebrafish were exposed to a concentration series of each chemical (0–50 μM) in rotating sealed glass vials to minimize volatilization. Benchmark concentration (BMC_50_) values were calculated for morphological endpoints and lowest effect levels were determined for behavioral effects. The data were assessed for evidence of a narcotic mode of action using body burden measurements for select chemicals and logK_ow_ modeling. Targeted transcriptomics at a single concentration and timepoint as well as *in silico* molecular docking were conducted to generate mode of action hypotheses. The vial method enabled detection of highly variable developmental toxicity not previously observed using standard 96-well plate exposures. LogK_ow_ and body burden were poor predictors of toxicity, suggesting a non-narcotic mode of action. Transcriptomic analysis revealed evidence for the disruption of glucocorticoid signaling pathways. Molecular docking identified potential protein targets (e.g., CYP1A2, NT5E, FOLR1) that may mediate observed effects. This study demonstrates the importance of appropriate exposure methods for semi-volatile compounds, reveals structure-dependent toxicity among alkyl-substituted naphthalenes, and provides a foundation for further mechanistic studies and improved risk assessment of alkyl-substituted PAHs.

## 1. Introduction

Polycyclic aromatic hydrocarbons (PAHs) are a ubiquitous class of environmental contaminants characterized by two or more fused aromatic rings. PAHs are in high abundance in petroleum products and coal and are also produced through the incomplete combustion of organic materials like tobacco and wood (Casillas et al., 2025). While PAHs are naturally occurring chemicals, their presence in the environment is largely attributable to anthropogenic activities (Howsam & Jones, 1998). Naphthalene is the smallest of the PAH chemical class, comprised of two fused aromatic rings. Naphthalene has numerous industrial applications. Isolated from petroleum and coal tar, it is used in the production of polyvinyl chloride (PVC) plastics, dyes, resins, and insect repellent (Preuss et al., 2003). As a member of the US Environmental Protection Agency’s 16 priority PAH list, naphthalene has been included in many environmental monitoring studies (Hudson-Hanley et al., 2021; Keith, 2015; Souza et al., 2023). One study, using data collected from the National Health and Nutrition Examination Survey, measured the presence of oxygenated metabolites of four parent PAHs, including naphthalene in urine (Hudson-Hanley et al., 2021). Metabolites of naphthalene were 20–30 % more prevalent than the other PAHs measured and increased over the fourteen-year study (Hudson-Hanley et al., 2021). Environmental monitoring has routinely found that naphthalene is the most abundant PAH in air and water samples (Moran et al., 2023; Yu et al., 2021). While alkyl-substituted PAHs are much less commonly monitored, when measured, they are routinely more abundant than parent or otherwise substituted PAHs (Golzadeh et al., 2021; Moradi et al., 2022; Qiao et al., 2020; Wnorowski et al., 2022). Alkyl-substituted naphthalenes are typically the most abundant amongst alkyl PAHs (Peng et al., 2023; Wnorowski et al., 2022). For example, comprehensive review of PAH derivatives found that 2,3- and 2,6-dimethylnaphthalene alone made up 10 % of all alkyl PAHs measured (Peng et al., 2023).

There is a large body of knowledge concerning the toxicity of naphthalene, while research on alkyl-substituted naphthalenes, beyond 1- and 2-methylnaphthalene, is limited. Naphthalene was designated by the National Toxicology Program (NTP) as *reasonably anticipated to be a human carcinogen* in 2004 thus, carcinogenicity is typically the main concern for human health. Naphthalene can also cause respiratory and hepatic injury in a club cell specific manner (Chen et al., 2018; Van Winkle et al., 1997). The mechanisms underlying these effects are linked to the *in-situ* production of toxic metabolites (eg. Naphthalene 1,2-epoxide) by non-AHR induced cytochrome P450s (CYPs) (Casillas et al., 2025; Genter et al., 2006; Warren et al., 1982). Monomethylated naphthalenes are singly alkylated with similar production sources and uses and are associated with similar health effects as unsubstituted naphthalene (Casillas et al., 2025). The mechanisms underlying these effects are also linked to toxic metabolite related oxidative stress. Though ring oxidation leading to epoxide formation does occur with these chemicals, oxidation at the methyl group is the primary metabolic pathway (Casillas et al., 2025; Wang et al., 2020). Thus, any differences in toxicity between naphthalene and monomethylated naphthalenes are often attributed to the reduction in the formation of reactive epoxides (Höke & Zellerhoff, 1998). However, evidence suggests that 2-methylnaphthalene may also act through non-CYP dependent mechanisms, as CYP inhibition is not as effective in reducing toxicity as it is for naphthalene (Lin et al., 2009).

There is limited research on the toxicity of alkyl-substituted naphthalene compounds beyond monomethylated derivatives. In mice, 2-isopropylnaphthalene and 2,6-diidopropylnaphthalene are minimally active compared to naphthalene and its monomethylated derivatives (Honda et al., 1990). This difference in activity was attributed to the further reduction in ring oxidation due to the increased alkyl substitution of these compounds, and minimal formation of reactive epoxides (Höke & Zellerhoff, 1998; Honda et al., 1987; Kojima et al., 1982). Metabolite formation of a number of other alkylated naphthalenes beyond those previously mentioned, was conduct by Wang et al. (2020), who found that alkylated derivatives of naphthalene favored side chain oxidation over ring oxidation with negligible ring oxidation on naphthalenes with six or more alkyl substitutions (Wang et al., 2020). The authors suggested that the reduction in ring oxidation of more alkylated naphthalenes would equate to a reduction in bioactivation and toxicity (Wang et al., 2020). This hypothesis is yet to be confirmed *in vivo*. Another frequently proposed hypothesis for the activity of PAHs and alkyl-substituted PAHs more generally is the narcosis model. In it, chemicals act through baseline toxicity induced by basic partitioning into membranes, leading to membrane disruption (Klüver et al., 2016, 2019; Lambert et al., 2022). There is little experimental work that has tested these hypotheses.

The only prior large scale screenings of alkyl-substituted naphthalenes for vertebrate biological activity were our previous publications (Geier et al., 2018a; Morshead et al., 2025; Shankar et al., 2019). In these studies, 24 alkyl-substituted naphthalenes were screened for biological activity in early life stage zebrafish in a high throughput, 96-well plate format (Geier et al., 2018a; Morshead et al., 2025; Shankar et al., 2019). Embryos were statically exposed in 100 μL of exposure medium and sealed at 6 h post fertilization (hpf) until morphological and behavioral data were collected at 24 and 120 hpf (Geier et al., 2018a; Morshead et al., 2025; Shankar et al., 2019). The study showed no morphological effects and only minimal behavioral effects (Geier et al., 2018a; Morshead et al., 2025). Chemical fate analysis and modeling in the 96-well plate system determined that volatilization was the primary explanation for the minimal uptake of naphthalene by embryos (Geier et al., 2018b; Rude et al., 2025). The challenge of volatilization is not unique to naphthalenes in our previous exposure system, and has been documented with other semi-volatile and volatile compounds in diverse aqueous exposure scenarios (Jochum et al., 2024; Proença et al., 2021; Stadnicka-Michalak et al., 2021). We hypothesized that a more tightly sealed approach to developmental exposures would reduce volatilization and present a truer picture of alkyl naphthalene bioactivity (Geier et al., 2018a; Morshead et al., 2025).

Here we tested our hypothesis in rotating 2 mL glass vials with lined screw caps filled with 2 mL of exposure medium. The technique limited headspace and sealed much more effectively than a 96 well plate could be sealed. We screened naphthalene and the 24 alkyl-substituted derivatives tested in our previous 96-well plate studies for developmental toxicity in zebrafish. The data were used to assess whether increased ring oxidation equated to reduced toxicity, as previously proposed (Wang et al., 2020). We assessed whether alkyl-substituted naphthalenes acted through narcosis by assessing how well logK_ow_ correlated with the observed potency, as previous narcosis models have done (Di Toro et al., 2000; Klüver et al., 2016, 2019). To support our modeling work, body burden concentrations were measured in a subset of chemicals and compared to bioactivity. Targeted transcriptomics and reverse molecular docking data generated hypotheses for the mechanisms underlying the variability in potency of bioactivity. Transcriptomic responses were assessed for gene expression correlation with morphological effects. Reverse molecular docking to a library of ~30,000 predicted human protein binding sites enabled hypotheses about molecular initiating events. Proteins of interest were further assessed utilizing crystal structures and zebrafish protein structure predictions.

## 2. Methods

All data analysis, unless otherwise stated, was performed in R studio (R version 4.4.2) (R Core Team, 2023).

### 2.1. Chemicals

Analytical grade standards were obtained from the Chemical Mixtures Core of the Oregon State University Superfund Center from various vendors (Table S1). All standards were analytically verified and dissolved in dimethyl sulfoxide (DMSO) if obtained neat or solvent exchanged to DMSO (10 mM concentration). A detailed chemical list with CASRN and stock concentration can be found in Supplemental Information 1 (SI-T1).

### 2.2. Zebrafish husbandry

Adult tropical 5D line zebrafish (*Danio rerio)* were maintained under Institutional Animal Care and Use Committee protocol 2021–0227 at the Oregon State University, Sinnhuber Aquatic Research Laboratory (SARL, Corvallis OR). Fish were kept in densities of ~ 500 fish/35-gallon tank at 28 °C in a 14 h:10-hour light:dark cycle, tank water was recirculated, filtered water was supplemented with Instant Ocean salts. Adult fish were fed twice a day with Sparos Zebrafeed 300 μm and spawned by placing a specialized gridded platform and funnel in the tank the night before, spawning was initiated upon first light in the morning. The resulting embryos were collected, screened for malformed or unfertilized individuals, and sorted to the same developmental stage into large glass petri dishes containing embryo medium (EM) (Kimmel et al., 1995).

### 2.3. Toxicity testing

**Fig. 1.**
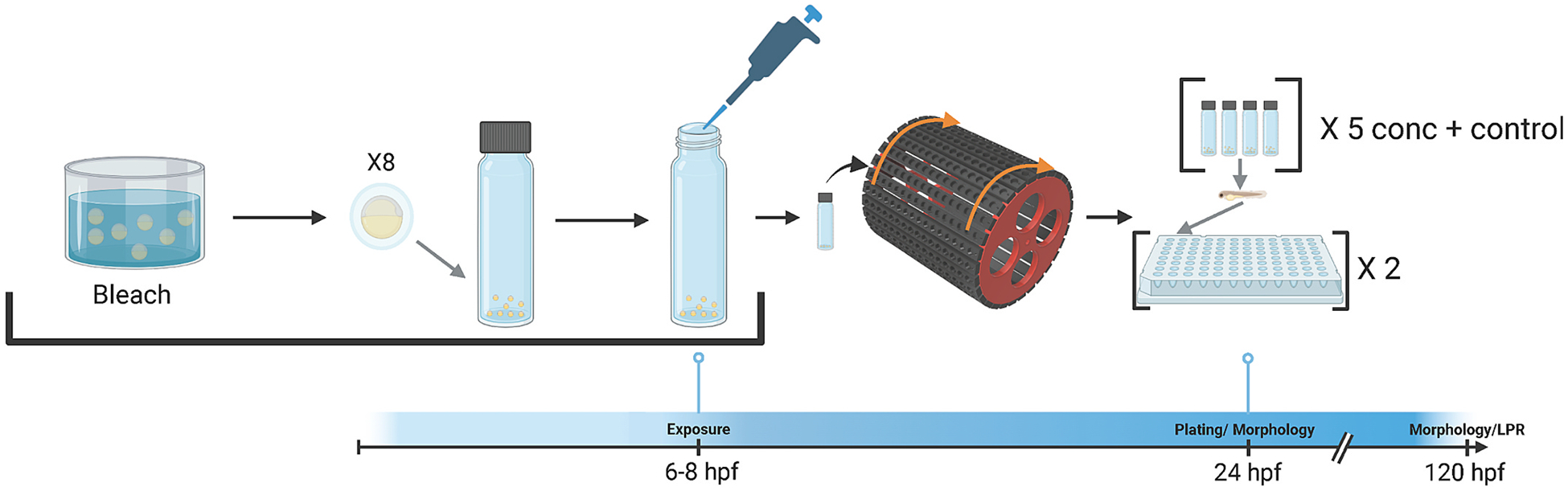
Exposure Method. Vial exposure method designed for high-throughput developmental zebrafish exposures of semi-volatile compounds.

When embryos were 6 hpf they were bleached in groups of 1000 or less to limit bacterial growth in sealed vials. Embryos were placed in a strainer for transfer between solutions in 500 mL containers. First embryos were placed in system fish water for 1 min, then transferred to a 2.25 % sodium hypochlorite solution for five min, then transferred to a 70 ng/mL sodium thiosulfate solution for 1 min and rinsed in EM for five min and transferred to a clean glass petri dish. Pools of eight embryos were placed into 2 mL clear glass screw cap exposure vials with polyvinyl-faced pulp liner (Avantar Radnor, PA, United States) using a glass Pasteur pipette. Excess EM was removed, and 2 mL of fresh EM was immediately added. A total of 10 uL of 200x working stock (in DMSO) was added to each vial, then immediately capped and inverted three times to mix. No precipitation was observed for any chemicals at the concentrations tested. Vials were loaded into a custom 3D printed vial holder for slow end-over-end rotation in a dark 28 °C incubator overnight. Rotation of the vials served to reduce chemical partitioning into the headspace and ensure uniform respiration of embryos (Bedell et al., 2025). At 24 hpf, embryos were removed from exposure vials and singulated into 96-well plates loaded with 100 μL chemical-free EM. Each exposure vial group was maintained in plate columns. After loading into 96-well plates, embryos were assessed for 24-hour mortality. For the remainder of the exposure, plates were kept stationary at 28 °C in the dark, until 120 hpf when morphology screening and the larval photo motor response (LPR) assay were conducted, section 2.3.2 and section 2.3.3, respectively. 96-well plates were labeled with a unique plate identifier and all data collected for the endpoints explained in the following sections were connected through a custom laboratory information management system (Zebrafish Acquisition and Analysis Program (ZAAP)) (Truong et al., 2016). A visual summary of this exposure method is shown in Fig. 1.

#### 2.3.1. Experimental design

All chemicals were tested at five concentrations and a control (0, 3.125, 6.25, 12.5, 25, 50 μM) and normalized to 0.5 % DMSO, a concentration that did not cause morphological effects but fostered solubility (Hoyberghs et al., 2021). Each treatment group was replicated over four vials, resulting in 32 embryos per treatment, split over two replicate 96-well plates per chemical. Some chemicals lacked intermediate response data (between background and 100 % effect) which hindered our ability to accurately model the sigmoidal concentration-response curve and estimate benchmark concentration 50 % (BMC_50_) values. For these chemicals, follow-up exposures were conducted at four intermediate concentrations between background and 100 % effect to improve sigmoidal concentration–response modeling. These concentrations were 12.8, 16, 20, and 25 μM for chemicals lacking data between 12.5 and 25 μM and 30, 37.5, 45, and 50 μM for chemicals lacking data between 25 and 50 μM. Vial groups of plated larvae were tracked to assess the impact of the vial on the results. All reported concentrations were nominal water concentrations and are included in SI-T4.

#### 2.3.2. Morphology

At 24 hpf embryos were assessed for mortality (MO24). At 120 hpf embryos were assessed for abnormality in nine morphological endpoints: mortality (MORT), edemas (EDEM), bent axis (AXIS), touch response (TCHR), craniofacial (CRAN), muscular/cardiovascular (MUSC), lower trunk (LRTK), brain (BRAN), skin (SKIN), and notochord malformations (NC). Morphological assessments were made by a team of three to four individuals. The team was blinded to the identity, plate layout, and concentrations of the chemicals, thereby minimizing bias in morphological screening assessment. To ensure the assessments were consistent, concentration–response reproducibility of a parathion ethyl quality control plate was performed along with each experimental exposure set up. Details of all measured endpoints can be found at Developmental toxicity zebrafish phenotype atlas. The binning of morphological endpoints for assessment was done as previously described in (Wilson et al., 2022). Each endpoint was scored as a binary presence/absence and scores were compiled into an “Any effect” endpoint, the presence of abnormality in any individual endpoint.

##### 2.3.2.1. Vial effect assessment.

Morphological results were assessed for the impact of vial exposure groups on morphological outcomes. This assessment used generalized linear mixed effects models and variance source partitioning for each chemical using lme4::glmer() function in R (Bates et al., 2015). Scaled concentration (log-plus-one scaled) was used as the fixed effect to predict the binomial presence or absence of the Any effect endpoint and the vial group of a given observation was assigned as the random effect. R^2^ values for the model fixed effect (concentration) and random effect (vial group) for each model were obtained using the partR2::partR2() function, remaining error was calculated as 1-fixed R^2^-random R^2^. The ratio of fixed R^2^:random R^2^ was calculated, a ratio > 1 indicated that the impact of the vial grouping explained less of the variability observed in the data than the concentration, thus it was appropriate to treat embryos as individual observations. For chemicals with a fixed R^2^:random R^2^ ratio < 1 the ratio of random R^2^:remaining error was used to determine the magnitude of variability explained by the vial groupings. Low random R^2^:remaining error ratios (<1) indicated that the amount of variability explained by the vial groupings was negligible in explaining the overall observed variability in the data and thus it was appropriate to treat embryos as individual observations for the following analysis. The result of this analysis is available in SI-T2. All chemical data sets met the criteria to treat embryos as individual observations for all downstream analysis.

##### 2.3.2.2. Morphology summary statistics.

The Any effect percent was used to calculate the BMC_50_, 50 % change relative to the background for each endpoint and chemical. All values were reported as the nominal exposure concentration. BMC_50_ values were calculated as previously described (Truong et al., 2022). Briefly, a unrestricted three-parameter log-logistic model for dichotomous data with “extra risk” was fit following guidelines described in the EPA BMDS v3.2 manual, using the maximum likelihood estimation to find the three parameters (US Environmental Protection Agency (EPA), 2020). These curves were used to calculate the reported BMC_50_ values which were required to be within the exposure concentration range, 95 % confidence intervals (BMC upper (BMCu) and BMC lower (BMCl)) were computed using the likelihood-ratio method described in the EPA BMDS v3.2 manual (US Environmental Protection Agency (EPA), 2020). BMC values were reported if the BMCu:BMCl ratio was < 40. BMC_50_, BMCl and BMCu and binomial response data are available in SI-T3 and SI-T4, respectively.

##### 2.3.2.3. LogK_ow_ modeling.

Average predicted logK_ow_ values for each chemical were obtained from the Comptox dashboard (version 2.5.2) when available (Williams et al., 2017). The logK_ow_ value for 2,4,5-trimethylnaphthalene was unavailable on the Comptox dashboard and was predicted using the KOWWIN module in EPI suite^™^Estimation Programs Interface (US EPA, 2024). The BMC_50_ values for chemicals with calculated values for the Any effect endpoint and predicted logK_ow_ values were used to build a simple linear model in R. Model performance was assessed using p-value, R^2^, and average relative error. The average relative error was calculated for each chemical in the model using the equation, *|residual|/observed BMD*_*50*_ and averaged. Model parameters are available in Table S2 and predicted logK_ow_ values are included in SI-T5.

#### 2.3.3. Larval photomotor response (LPR)

The LPR assay was conducted at 120 hpf. This was done in 96-well plates using a light–dark cycle and video tracking software via the Viewpoint ZebraBox behavioral system software version 5.29.0.70 (Viewpoint Life Sciences, Lyon, France). Larval movement was tracked throughout minutes 6–24. Animals dead or morphologically abnormal were excluded from the analysis, and if more than 30 % of the wells in a treatment group were excluded, the treatment was removed.

##### 2.3.3.1. LPR summary statistics.

Animal removal due to mortality and morphology effects precluded estimation of concentration–response curves, instead the lowest effect level (LEL) was used. Movement values were binned into 6s intervals, log normalized and used to calculate area under the curve (AUC). AUC values from the first cycle after calibration were used to calculate the LEL for each period (light and dark). AUC values were compared to the day-matched control using a Student’s *t*-test (Holm-corrected p value ≤ 0.05). An LEL value was only reported where the next highest concentration also showed a significant effect in the same direction, except for the highest concentration. This minimized the risk of false positives. LEL values and individual animal movement data are available in SI-T6 and SI-T7 respectively.

### 2.4. Body burden measurements

Body burden was measured for exposure to five chemicals at two timepoints: 24 and 48 hpf. These chemicals (1,5-dimethylnaphthalene, 1,7-dimethylnaphthalene, 2,7-dimethylnaphthalene, 2,4,5-dimethylnaphthalene, and naphthalene) were chosen to span a range of chemical toxicity based on morphological BMC_50_ values and to draw comparisons between chemicals with similar structures and variable toxicity. The exposure concentration was chosen as it was the maximum possible concentration that ensured enough viable animals for collection at 48 hpf and allowed direct comparison of chemical uptake at the same exposure concentration.

#### 2.4.1. Tissue collection

Chemicals were exposed at 20 μM following the exposure procedure detailed in section 1.3, 24 vials per chemical. At 24 hpf, embryos were pooled in groups of 32 embryos (combining embryos from 4 vials) into 1.5 mL polypropylene RINO-safe lock tubes (n = 3 per chemical treatment, n = 3 per control). Tubes were briefly placed on ice to reduce embryo movement (<1 min); the remaining medium was removed; the tube was then flash frozen in liquid nitrogen and stored at −20 °C until extraction. Embryos in the remaining vials were plated as previously described in section 1.3 until collection at 48 hpf following the same procedure as 24 hpf collections (n = 3 per chemical treatment, n = 1 control).

#### 2.4.2. Tissue extraction

Tissue extraction was performed as previously reported (Rude et al., 2025). Each sample was spiked with 25 μL internal standard in ethyl acetate and 100 μL of 1 mm glass beads (Next Advance, Troy, NY United States) were added to the RINO-safe lock tubes containing thawed tissue samples and left on ice for 10 min 250 mg of Na_2_SO_4_ and 200 μL of ethyl acetate were added to each tube and vortexed. Tubes were homogenized at speed 8 for 6 min in a Bullet Blender (Next Advance, Troy, NY, United States). 300 μL of ethyl acetate was added to each tube and the homogenization step was repeated until no pieces of non-homogenized fish tissue remained. Samples were then centrifuged for 10 min at 4 °C, 1600g. Sample supernatant (0.5 mL) was collected into amber vials and stored at 4 °C until chemical analysis.

#### 2.4.3. Chemical analysis

Samples were analyzed for quantitation and identification of 161 PAHs. The method was performed with an Agilent 7890 gas chromatograph (GC), with a 7000C triple quadrupole mass spectrometer (MS/MS) using an Agilent J&W PAH select (30 m × 250 μm × 0.15 μm GC column). Calibration for each analyte utilized at least a 4-point (range: 4–7, median 6 points, n = 161) curve with R^2^ ≥ 0.99. Specific instrument conditions were modified from Anderson et al. (2015) and are detailed in Table S3. GC–MS/MS data was analyzed using MassHunter Quantitative Analysis v.B.06.00 SP1 build 6.0.388.1 software (Agilent Corp. Wilmington,DE) (Anderson et al., 2015). Dynamic muti-reaction monitoring mode was used during analyses, and all PAHs were identified using analytical standards obtained from multiple sources (Table S1). Collison energies were optimized for each individual unsubstituted PAH, alkylation series (C1, C2, etc.), and when possible, specific alkylation group (i.e. methyl, ethyl, etc.).

A standard containing all 161 target analytes was used for continuing calibration verification (CCV) and was analyzed before and after each batch of samples with additional checks for system performance approximately every 10 samples. The data quality objective of at least 80 % of the 161 PAHs (i.e. 129 compounds) within 30 % of the expected concentration was met for all CCVs. In addition, specific PAHs used for exposures were all within 30 % of the known value. Solvent blanks were analyzed after every fifth sample and after each CCV, and all target PAHs were below detection limits. PAHs not used for exposures were below detection limits in all samples. To demonstrate precision, one extract was analyzed in duplicate; duplicate analysis displayed an average relative percent difference (RPD) of 0.7 % across detected PAHs. Finally, as a check against potential matrix influence regarding accuracy, one sample extract was over-spiked with a solution containing all 161 PAHs, and the resulting average percent recovery was 104 % with a range of 78–123 %. Resulting values are available in SI-T8.

#### 2.4.4. Statistical analysis

Body burden concentrations based on average embryo wet weight at each timepoint were determined by the equation below. Total measured chemical was calculated from ng/mL concentrations times sample volume (0.5 mL).

$$\text{Bodyburden}\;\left(\text{nmol}/\text{mgbw}\right)=\frac{\text{total measured chemical}\;\left(\text{nmol}\right)}{\text{number of fish}\left(32\right)\times \text{average body weight}\;\left(mg\right)}$$

Average body weights at 24 hpf and 48 hpf were assumed to be 0.31 mg and 0.26 mg, respectively as determined previously (Rericha et al., 2024). Statistical analysis to compare time points within chemicals was performed using a two-sided Student’s *t*-test (p-value ≤ 0.05). An ANOVA with a follow-up Dunn’s test was used to compare body burden between chemicals at 24 hpf (p-value ≤ 0.05). Body burden concentrations at 24 hpf were compared to the estimated percent effect of each measured chemical at the exposure concentration used for body burden analysis (20 μM). These estimates were made using the fitted concentration-response curves previously described in section 2.3.2.2.

The concentration uptake ratio of naphthalene at 24 and 48 hpf (nmol/embryo)/(nmol of nominal chemical exposure), was compared to the 96-well exposure method using previously published results (Geier et al., 2018b; Rude et al., 2025). Concentration uptake ratios between vial exposures and 96-well plate exposures were compared using a two-sided Student’s *t*-test, p-values ≤ 0.05 were considered significant. Concentration data as well as metadata highlighting differences in experimental design are included in SI-T9.

### 2.5. Targeted transcriptomics

Targeted transcriptomics were performed using the Biospyder^™^ TempO-Seq^™^ 96-well plate platform. This platform used detector oligos for a gene set of 3112 probes selected through data driven approaches and expert curation (Balik-Meisner et al., 2019). The targeted transcriptomics approach was selected to maximize the number of chemicals that could be included in the analysis while managing cost and data analysis requirements. Targeted transcriptomics were limited to 96 samples, three chemicals without morphological effects were excluded from the analysis to ensure n = 4 per chemical and n = 6 controls, excluded chemicals are indicated in SI-T3.

#### 2.5.1. Exposures and RNA collection

A single exposure concentration of 20 μM was chosen as the maximum possible concentration to elicit a biological response that also ensured enough viable animals for RNA collection for every chemical. The same concentration per chemical was chosen as opposed to a phenotypically anchored concentration due to variability in 48 hpf survival across chemicals at the same 120 hpf percent effect and to allow for a direct comparison of chemical potency and transcriptomic response. 48 hpf was chosen to ensure the measurement of transcriptomic responses prior to the emergence of observable phenotypic effects. This timepoint has been used extensively and enhanced the ability to compare our results across datasets. Exposure was conducted over two days, chemicals were split into day groups by numerical order of randomly generated internal labels, 11 chemicals and control each day, n = 48 embryos across six vials per treatment. Exposure was conducted as previously described in section 2.3. At 48 hpf embryos were collected for RNA extraction. Embryos from two exposure vials were kept for morphological effect assessments which were as expected based on screening results. From the remaining wells, six phenotypically normal embryos were collected and pooled in their vial groups into 1.5 mL Eppendorf tubes, placed on ice (~30 sec), excess medium was removed, placed back on ice (~30 sec), and 200 μL RNA shield was added. Five samples of n = 6 pooled embryos were collected from each treatment.

#### 2.5.2. RNA extraction and quantification

RNA was extracted from exposure groups on day one and day two. 100 μL of lysis buffer was added to each tube containing pooled embryo samples and 200 μL of RNA shield, the full contents of the tube were then transferred to the homogenization plate (2 mL deep 96-well plate pre-filled with 400 μL 1 mm zirconium beads and 500 μL of lysis buffer). The plate was homogenized for 1.5 min using the Mini-BeadBeater 96 (BioSpec Products, Bartlesville, OK, United States) and centrifuged for five min at 3000 rcf. 550 μL of the supernatant was distributed over two plates, each pre-filled with 275 μL of ethanol. RNA extraction was performed using the KingFisher Apex System (ThermoFisher Scientific, Waltham, MA, United States) and *Quick*-RNA Magbead kit (Zymo Research Corporation, Irvine, CA, Untied States). Briefly, this automated process uses Mag beads to collect RNA from the sample, after a series of washes and treatment with DNase the RNA is eluted in 50 μL of ultra-pure water. RNA quality was assessed using the 4150 TapeStation System (Agilent, Santa Clara, CA, United States). All samples used had RNA integrity number (RIN) scores >9.8. RNA concentration was determined using Quant-iT RNA Reagent and assay kit and Varioskan ALF multi-mode plate reader (ThermoFisher Scientific, Waltham, MA, United States) in duplicate, samples with high variability (coefficient of variance (CV) > 10) between reads were repeated in triplicate. The average of the quantification reads was used for RNA concentration normalization. Of the five replicates, samples with the highest concentration and lowest CV were selected (n = 4 per chemical treatment n = 3 per day match control). All 94 samples were normalized using ultra-pure water to the lowest concentration sample − 14.9 ng/μL.

#### 2.5.3. Library preparation and sequencing

1 μL per sample of normalized RNA or ultra-pure water for two blank wells, was used as input for library preparation following the manufacturers protocol for the 96-sample TempO-Seq S1500 + Zebrafish Surrogate Assay panel (BioSpyder Technologies, Inc., Carlsbad, CA, United States). Briefly, samples were added to the assay plate with an equal volume of 2x enhanced lysis buffer and processed through annealing with detector oligos, digestion, and ligation. Proper amplification was verified using real-time PCR green fluorescence. Library purification was performed using the NucleoSpin Gel and PCR Cleanup Kit (Macherey-Nagel Inc., Allantown, PA, United States) following adjustments detailed in the manufacturer’s instructions. PCR amplification products were pooled into a single sequencing library of 5 μL each into a 1.5 mL Eppendorf tube. The pooled and purified libraries were sequenced using one lane of the NovaseqX 10B flow cell (BioSpyder Technologies, Inc., Carlsbad, CA, United States).

#### 2.5.4. Data analysis

##### 2.5.4.1. Pre-processing.

Demultiplexing and alignment was performed using the manufacturer’s platform TempO-SeqR (BioSpyder Technologies, Inc., Carlsbad, CA, United States). The output table of gene counts was used for downstream analysis. Total reads per sample ranged from 7.4 million to 16.8 million with a mean of 10.6 million reads. Sample naming metadata and raw read counts are available in SI-T10 and SI-T11, respectively. All samples passed quality control metrics where sequence depth > 300 K, >40 % reads aligned to probe, >30 % of reads aligned uniquely to the probes, >300 K aligned reads, >30 % of the probes had at least five reads, per base sequence quality was high, and per base N content was low. Counts of genes with multiple probes were summed as instructed by the manufacturer resulting in 3069 unique genes (Everett et al., 2022). Read counts were normalized by upper quartile normalization using the *calcNormFactors* function from the Bioconductor package in EdgeR with the 75th percentile of reads per samples as the scaling factor. This normalization was chosen following recommendations for TempO-Seq data (Bushel et al., 2020). Principal component analysis (PCA) of normalized read counts identified a control sample from day one as an outlier which was removed from future analysis. PCA was used to determine that a batch effect was present based on exposure and RNA extraction day (nested by experimental design). The batch effect was removed using the *ComBat_seq* function from the SVA R package as previously applied to data of this type (Lee et al., 2024). Briefly, this function applied a negative binomial regression model to estimate and remove batch effects from count data (Zhang et al., 2020). This function worked with unnormalized read counts and produced batch corrected but not normalized counts. Upper quartile normalization previously described was applied following batch correction. Normalized and batch corrected read count data are available in SI-T12. A PCA was used to check the results of the batch correction (Fig. S1). Targeted transcriptomic data are available through NCBI’s Gene Expression Omnibus accession number GSE296410 (Edgar et al., 2002).

##### 2.5.4.2. Differential expression analysis.

Differential expression analysis between controls was performed to determine if a global control would be appropriate. Differential expression analysis was done using the limma package by fitting linear models with empirical Bayes smoothing. P-value was adjusted using the Benjamini-Hochberg method and the adjusted p-value was used for all analysis (Ritchie et al., 2015). There were no differentially expressed genes (DEGs) between the day-matched controls (p-value ≤ 0.05) therefore, a global control was used for analysis. DEGs for the chemical exposures were calculated using the method described above compared to global controls (p-value ≤ 0.05 and log_2_ fold change (log_2_ FC) ≥ |1|) (SI-T13).

##### 2.5.4.3. Correlation analysis.

Correlation analysis was performed to examine the relationship between the log_2_FC of the top five most frequently differentially expressed genes (DEGs in at least 25 % chemical exposures) and morphological BMC_50_ values. This was done using a Spearman’s rank correlation analysis log_2_FC values for the five genes meeting these criteria and the BMC_50_ values (chemicals without a BMC_50_ value were assigned 50 μM). Spearman’s rank correlation was used as opposed to Pearson’s to include chemicals without a calculated BMC_50_ value. Correlations were considered significant if Holm-adjusted p-value ≤ 0.05 and rho (ρ) ≥ 0.5, results available in Table S4.

### 2.6. Reverse molecular docking

Naphthalene and alkyl-substituted naphthalene structures were docked to predicted binding sites of AlphaFold2 predicted structures (Sim et al., 2023; Varadi et al., 2022). Description of the generation of the binding pocket list used in this screen was previously reported (Kong et al., 2024). Briefly, binding pocket predictions were acquired from the HPRoteome-Bsite database (Sim et al., 2023) which contains binding pockets for 16,554 proteins covering 80 % of the UniProt entries in the AlphaFold human proteome database (Varadi et al., 2022). While many proteins had one predicted domain, some had multiple. For each predicted protein domain, both the highest-ranking sequence-based and 3D-structure-based predictions were used in the screen, resulting in 33,446 total binding pockets. Reverse molecular docking screening and validation using zebrafish ortholog sequences were conducted with Autodock Vina v. 1.2.3. Naphthalene and alkyl-naphthalene ligand structures were generated by conversion of smiles sequences obtained from ChemDraw version 21.0.0 (Revvity Signals Software, Inc.) to pdbqt files using Open Babel (O’Boyle et al., 2011). For each protein, the value from the binding pocket and prediction method with the lowest binding energy value was selected for further analysis (all binding energy values available in SI-T14). For each chemical, the binding energy values for each protein were used to create a rank with 1 being the protein with the lowest binding energy value for each respective chemical. These ranks were compared as opposed to predicted binding energy values due to variability in mean binding energy of each chemical.

Follow up docking of NT5E was conducted using a zebrafish derived AlphaFold structural prediction and two experimentally derived structures of human NT5E. The two 3D crystal structures were obtained through the Protein Databank (PDB, www.rcsb.org); PDBIDs were 4H2I and 7P9N. Preparation of the structures for docking was done using Chimera. Ligands and solvents were removed during docking prep with default settings including metal ion charges (Zn^2+^, Ca^2+^, and Cl^1+^) (Pettersen et al., 2004). Docking was conducted using the AutoDock Vina plug-in. The docking boxes were chosen to encompass the area containing the experimental ligand (7P9N centroid: 9.575, 11.878, 36.011 size: 30 × 30 ×30 Å; 4H2I centroid: −18.924,21.495, −30.287, size: 18 ×18 × 16 Å).

The zebrafish-derived protein prediction (zf-Nt5e) was obtained from the AlphaFold protein structure database (alphafold.ebi.ac.uk/download). The binding site location was predicted using Proteins*Plus* (proteins.plus) and adenosine monophosphate (AMP) as the representative ligand (Centroid: 3.699, −1.229, 2.578, size: 20 × 20 × 20 Å) (Schöning-Stierand et al., 2022). Docking prep and docking were conducted following the same procedures as the two crystal structures. Follow-up docking scores can be found in SI-T15.

## 3. Results and discussion

### 3.1. Morphology and Behavior

The vial exposure method revealed morphological effects from alkyl-substituted naphthalene exposure that were not detected from 96-well static exposure screening in previous studies (Geier et al., 2018a; Morshead et al., 2025; Shankar et al., 2019). Vials were more effective for this set of semi-volatile compounds, and we would expect the same for other semi-volatile compounds. Morphological effects in controls were well below the 20 % quality control threshold used in our 96-well exposure method. Seven of the chemicals tested did not have an Any effect BMC_50_ value and those that did had values ranging from 46.6 μM to 13.8 μM (Fig. 1).

**Fig. 2.**
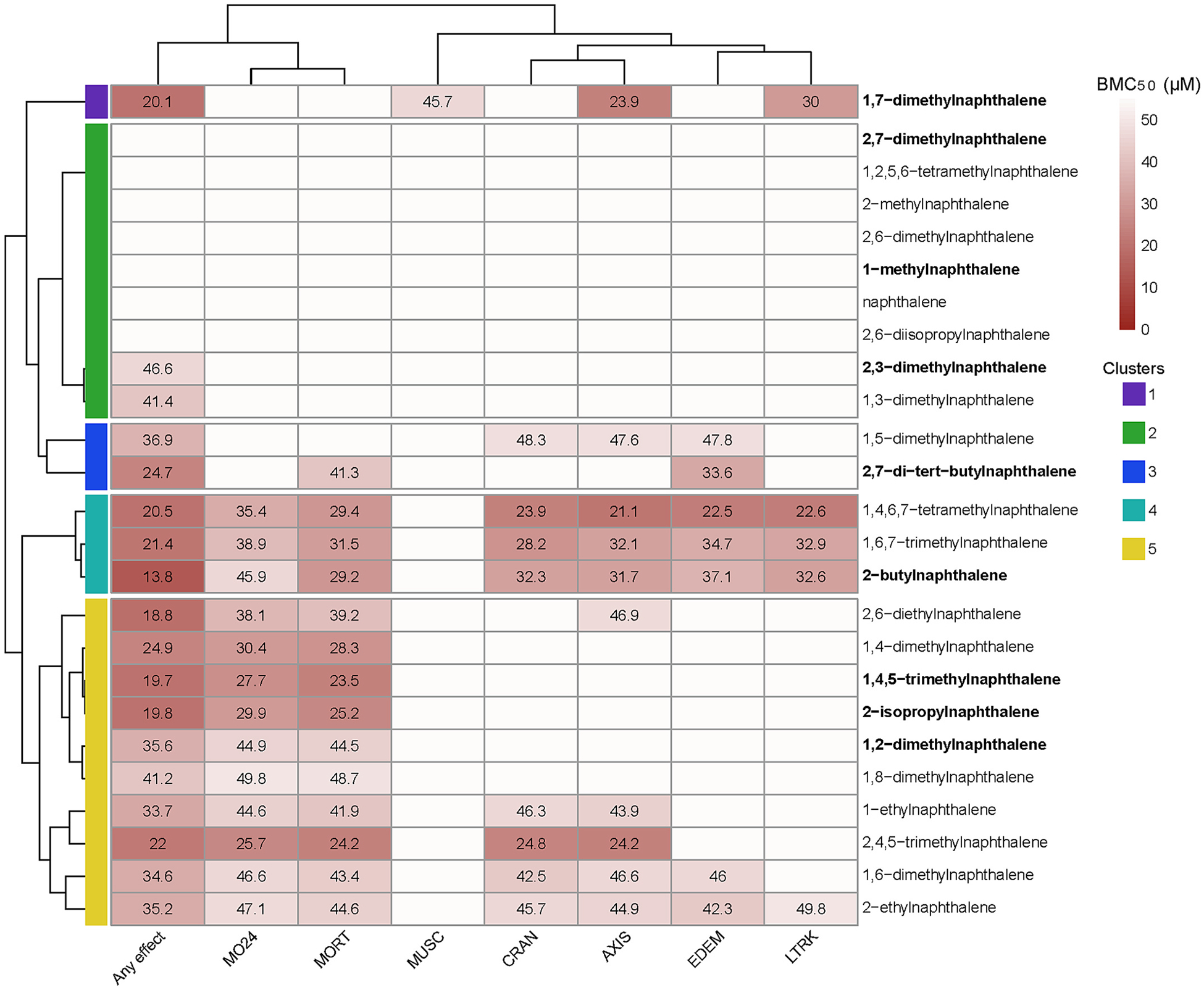
Morphological Screening Results. Heatmap displaying morphological Any effect benchmark concentration 50% (BMC_50_) values for all endpoints. Endpoints and chemicals were hierarchically clustered based on similarities. Darker red indicate a lower BMC_50_ value. Bold chemical names indicate chemicals with behavioral effects in the larval photomotor response (LPR) assay. Mortality at 24 hpf (MO24), mortality at 120 hpf (MORT), craniofacial malformation or missing eye, jaw, snout at 120 hpf (CRAN), curved or bent body axis at 120 hpf (AXIS), pericardial and/or yolk sac edema, heart and/or yolk sac malformation at 120 hpf (EDEM), lack of circulation, malformed or missing somites and/or swim bladder at 120 hpf (MUSC), malformed lower trunk and/or caudal fin at 120 hpf (LTRK), summary endpoint-if a fish had an instance of any of the above endpoints (Any effect).

The heatmap in Fig. 2 shows the BMC_50_ values for each chemical across all endpoints which had a BMC_50_ value for at least one chemical. MO24 and MORT clustered with Any effect while the non-mortality endpoints grouped together indicating that the Any effect endpoint was driven by mortality for many of the chemicals. Chemical clustering based on the phenotypic profiles revealed five distinct groupings. Cluster one contained only 1,7-dimethylnaphthalene, which had the lowest BMC_50_ value for Any effect of the dimethyl-substituted naphthalenes tested. This chemical had a unique morphological profile in that it was the only chemical with a BMC_50_ value for the MUSC endpoint and was not associated with mortality. Cluster two contained nine chemicals which had the lowest response, with the majority having no BMC_50_ value for any endpoint. The two chemicals in this cluster with BMC_50_ values for the Any effect endpoint were close to the maximum concentration tested (50 μM). Cluster three contained two chemicals, 1,5-dimethylnaphthanlene and 2,7-di-*tert*-butylnaphthalene which both had no MO24 BMC_50_ and BMC_50_ values for variable endpoints at 120 hpf. Cluster four chemicals (1,4,6,7-tetramethylnphthalene, 1,6,7-trimethylnaphthalene, and 2-butylnaphthalene) had the most diverse morphological effects with BMC_50_ values for all endpoints except MUSC. Cluster five contained 10 chemicals, all of which had BMC_50_ values for Any effect, MO24 and MORT and varying presence of activity in other endpoints (CRAN, AXIS, EDEM, and LTRK). 2-ethylnaphthalene specifically had less potent effects over the same endpoints as those in cluster 4.

Unsubstituted naphthalene as well as the monomethylated 1-methylnaphthalene and 2-methylnaphthalene all had no morphological BMC_50_ values for any morphological endpoints. While the majority of more highly alkyl-substituted naphthalenes caused morphological effects. Notably, 2,6-diisopropylnaphthalene did not; it was one of few alkyl-substituted naphthalenes previously tested in mice, and shown to cause minimal damage to lung tissue (Honda et al., 1990). The lack of activity for this chemical may indicate that reduction in ring oxidation in alkyl substituted naphthalenes reduces their toxicity (Höke and Zellerhoff, 1998; Lin et al., 2009; Wang et al., 2020). While our data for 2,6-diisopropylnaphthalene agrees with previously reported toxicity, our more extensive screen identified many alkyl substituted naphthalenes with potent toxicity. This suggests that lower ring oxidation among higher alkyl substituted species may not correlate with reduced toxicity or that their toxicity is not mediated through ring oxidation metabolites.

**Fig. 3.**
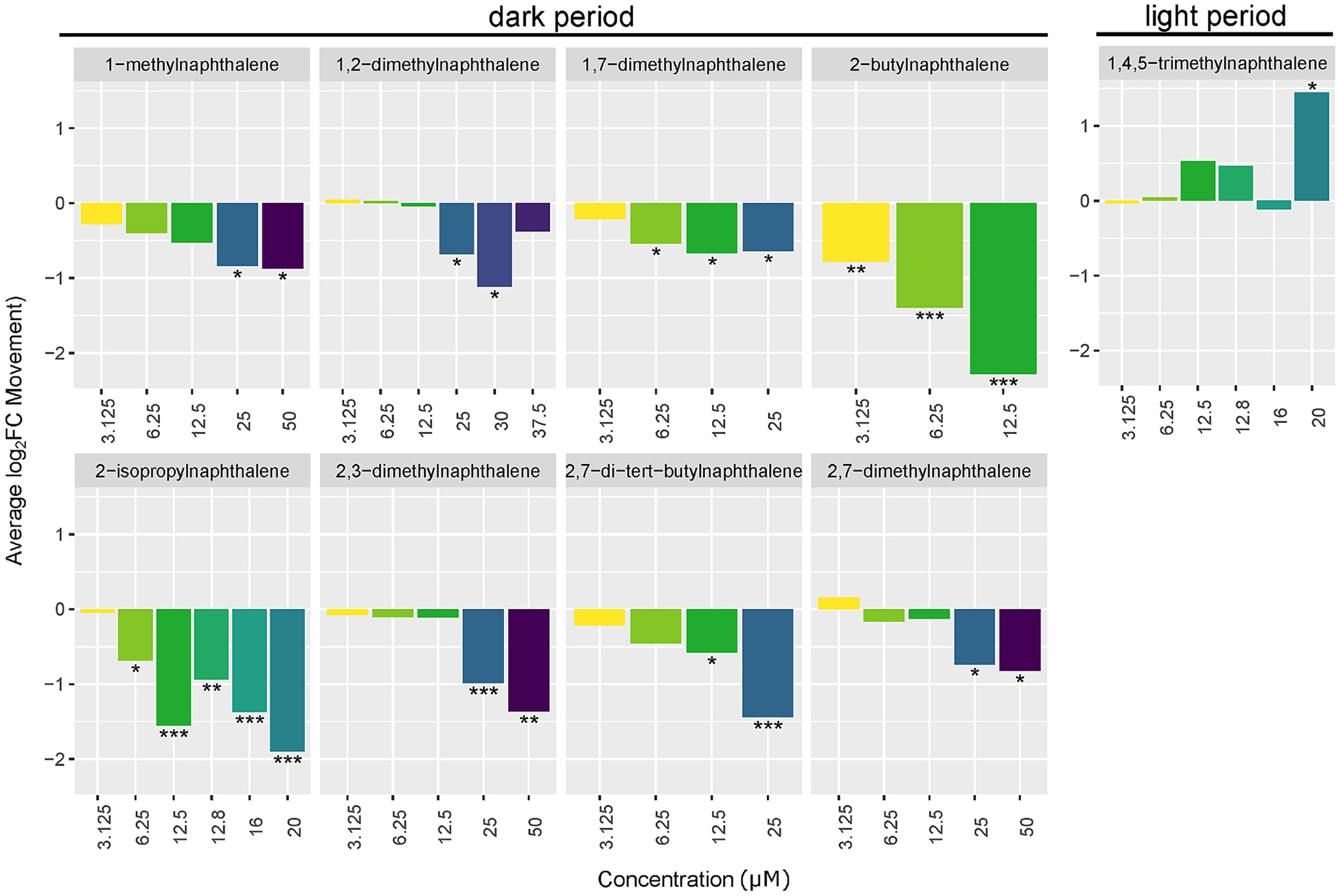
Larval Photomotor Screening Results. Average log_2_ fold change (log_2_ FC) movement from day-matched controls for chemicals with significant behavioral effects. Significance was determined by the lowest effect level (LEL), where the next highest concentration also showed a significant effect in the same direction—except for the highest concentration.

Chemicals with behavioral effects (bolded chemical names in Fig. 2) did not follow morphological effect groupings. Most LPR effects were hypoactivity in the dark period with increasing LPR effects at higher concentrations, apart from 1,2-dimethylnaphthalene which had the opposite trend (Fig. 3). One chemical, 1,4,5-trimethylnaphthalene, caused hyperactivity during the light period, which was only observed at the highest concentration (Fig. 3). Larval movement tracks over time for chemicals with LPR LEL values are shown in Fig. S2. Nine of the chemicals were previously screened in the 96-well plate format where all but 2,6-dimethylnaphthalene caused LPR effects (Geier et al., 2018a). Here seven of those previously screened were not active in the LPR assay, but 1-methynaphthalene and 1,2-methylnaphthalene were. This unexpected result was likely due to the more stringent criteria for behavioral activity used here which required that multiple consecutive concentrations have significant LPR effects for estimation of an LEL.

### 3.2. LogK_ow_ modeling

Predicted logK_ow_ values for all 25 chemicals and their BMC_50_ values were used to build a simple linear model for the BMC_50_ concentrations (Fig. S3). These models are commonly used to determine if adverse effects are due to narcosis (Di Toro et al., 2000; Klüver et al., 2016, 2019). Narcosis generally lowers biological activity via narcotic disruption of cell membrane functions; the potency of a narcotic increases as membrane partitioning increases (estimated using logK_ow_). It is important to note that chemicals without an estimated BMC_50_ value for Any effect were not used to build either of the models presented. The R-squared value for model A, containing all chemicals with a BMC_50_ value, was 0.21, indicating that logK_ow_ explained 21 % of the variability observed in the BMC_50_ values. Model A did not meet the significance criteria with p = 0.053 (Fig. S3A). The predicted logK_ow_ of 2,7-di-*tert*-butylnaphthalene was identified as an outlier by interquartile range. Removal of 2,7-di-*tert*-butylnaphthalene as linear model B (Fig. S3B) improved the model to an R-squared and p-value of 0.61 and 0.00021, respectively. The model B predicted logK_ow_ explained 61 % of the variability observed in the data. Additionally, the average relative error (the average percent error between the predicted and observed BMC_50_ values) was 17.3 %, driven by many chemicals having similar predicted logK_ow_ around 4.3 and highly variable Any effect BMC_50_ values (Fig. S3). The domain of applicability of model B is limited as it can only be used for alkyl-substituted naphthalenes with logK_ow_ between 4.23 and 5.13 for which there is an Any effect BMD_50_ value below 50 μM. These limitations make the model impractical as a risk assessment tool. Several chemicals with no Any effect BMC_50_ and therefore not used in modeling, had high logK_ow_ > 5 which further emphasized the poor correlation between chemical potency and logK_ow_. We concluded that logK_ow_ did not predict alkyl-substituted naphthalene potency and that narcosis likely does not drive their biological activity.

### 3.3. Body burden

Body burden measurement of select chemicals was also used to test the narcosis hypothesis and understand alkyl-substituted naphthalene fate. Measurements were made at 24 and 48 hpf for five chemicals selected for variable potency and structural comparisons. Exposures were done at 20 μM, the maximum with enough viable embryos for collection at 48 hpf.

**Fig. 4.**
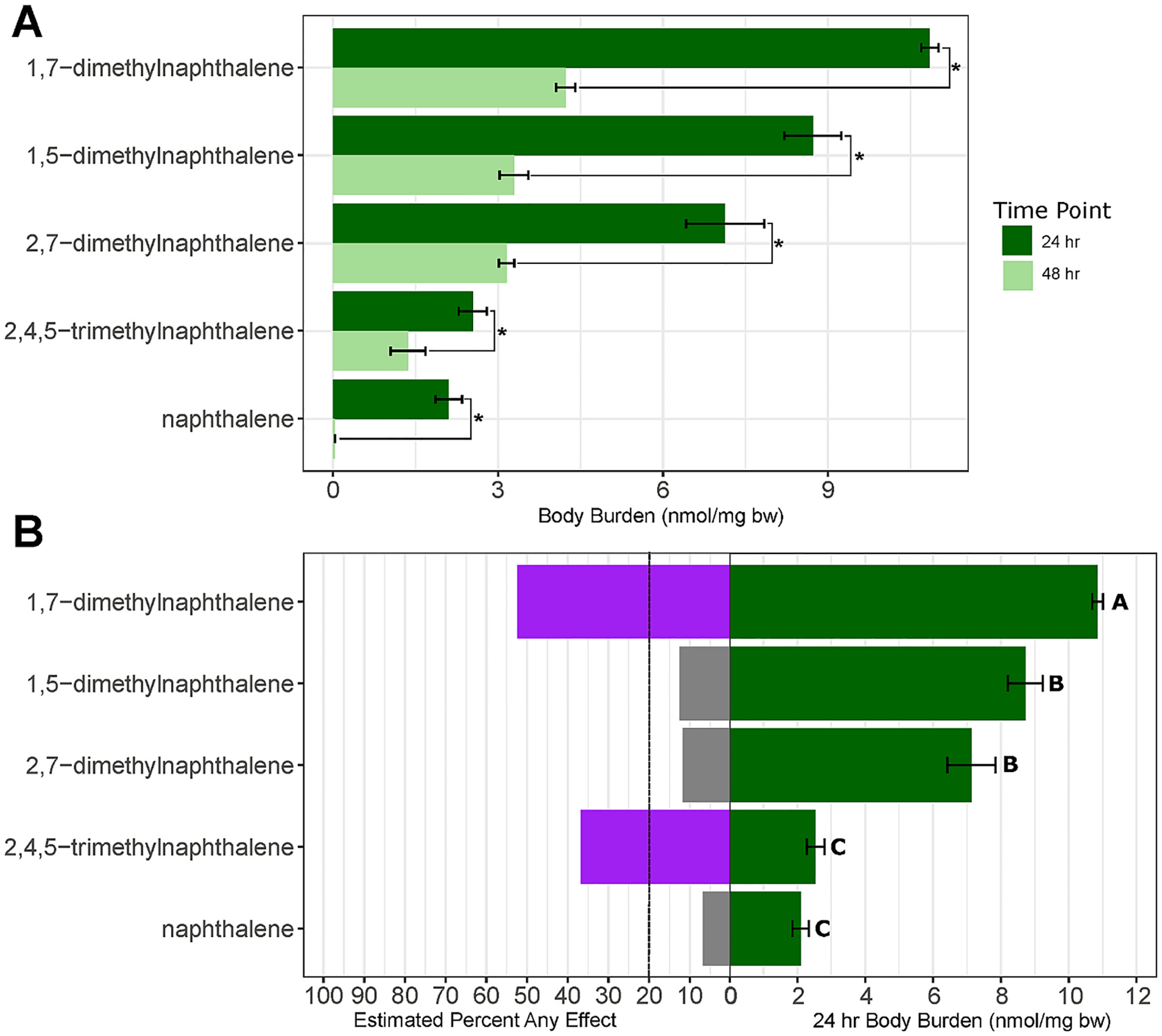
A. Body Burden Results. Bar plot of measured body burden (nmol/mg bw) at 20 μM of five naphthalenes at 24 and 48 hpf (n = 3). Error bars indicate one standard deviation from the mean. Timepoints were compared using a two-sided Student’s *t*-test, statistical significance indicated with asterisk (p-value ≤ 0.05) B. Bar plot comparing the measured body burden (nmol/mg bw) at 20 μM of five naphthalenes at 24 hpf to the estimated percent Any effect at 120 hpf. Estimated percent effect was calculated using dose–response curves. Error bars indicate one standard deviation from the mean. Significance determined using ANOVA with a follow-up Dunns test (p-value ≤ 0.05). Bars containing different letters were significantly different.

Body burden was significantly reduced for all chemicals from 24 to 48 hpf (Fig. 4A). In previous 96-well plate exposures, the body burden of naphthalene reached its maximum around 24 hpf. Though we did not have earlier time points to confirm, it was likely that body burden peaked around 24 hpf in vial exposures as well. In vial exposures the body burden loss by 48 h was 46 %-98 %, while in 96-well plate exposures most of the reduction in body burden occurred between 48 and 72 hpf, i.e. once metabolism became active (Rude et al., 2025). An earlier and more rapid reduction with the vial method was likely due to embryo removal from the chemical exposure at 24 hpf and singulation into chemical-free medium in plate wells.

The concentration uptake ratio of naphthalene (nmol/embryo per nmol available in EM) at 24 hpf in the vial exposures was 40x higher than in the 96-well exposure method 0.1303 vs. 0.0032 (Fig. S4). By 48 hpf the uptake ratio was slightly higher than the plate exposures of Geier et al. (2018b) (0.0013) but lower than the plate exposures of Rude et al. (2025) (0.0032) despite the large reduction between 24 and 48 hpf in the vial exposures (Fig. S4) (Geier et al., 2018b; Rude et al., 2025). This demonstrated the effectiveness of the vial exposure method in reducing chemical volatilization and increasing exposure concentrations.

While all chemicals followed the same trend over time, the body burden concentrations were highly variable at 24 hpf ranging from 2.10–10.6 nmol/mg bw. 1,7-dimethylnaphthalene had the highest concentration at 24 hpf followed by 1,5- and 2,7-dimethylnaphthalene, then 2,4,5-trimethylnaphthalene and naphthalene (Fig. 4B). Using body burden concentration instead of logK_ow_ as a proxy for membrane partitioning, we further tested the validity of the narcosis mode of action hypothesis. Body burden concentration at 24 hpf did not correlate with the estimated percent Any effect at 20 μM (from concentration-response curves described in Section 2.3.2; Spearman’s rank correlation p-value = 0.23 and ρ value of 0.7. The body burden of 2,4,5-trimethylnaphthalene was less than half that of 1,5- and 2,7-dimethylnaphthalene but had a much higher percent effect at 20 μM than either of the latter two chemicals (Fig. 4B). 2,4,5-trimethylnaphthalene had the lowest percent reduction in body burden between 24 and 48 hpf at 46 %. The moderate increase in 2,4,5-trimethylnaphthalene tissue persistence may have increased its toxicity but does not explain its toxicity relative to its reduced body burden. While 1,5- and 2,7-dimethylnaphthalene did have lower body burdens than 1,7-dimethylnaphthalene, this did not correspond to the differences in estimated percent Any effect (13 % and 12 % vs 52 % respectively). Incongruence between body burden and estimated percent Any effect further supported a non-narcotic mode of action.

### 3.4. Targeted transcriptomics

Transcriptomic data showed that separation along exposure/extraction day and batch correction, described in section 2.5.4.1, was successful in removing the apparent batch-based separation (Fig. S1). Based on PCA of batch corrected and normalized read counts, the overall data variability was small. Principal components one and two explained only 4.84 % of the variability. This suggested that few transcriptomic changes were captured in association with the chemical exposures. This may be due to the 20 μM concentration chosen to ensure sufficient larvae at 48 hpf. Based on our concentration-response curves, we predict a maximum effect of ~ 50 % for the most potent chemical at 120 hpf at 20 μM. When sampling RNA at only one timepoint there is the possibility of missing the strongest transcriptomic responses. It is also possible that disrupted pathways may not be represented in the genes included in the targeted transcriptomics platform.

**Fig. 5.**
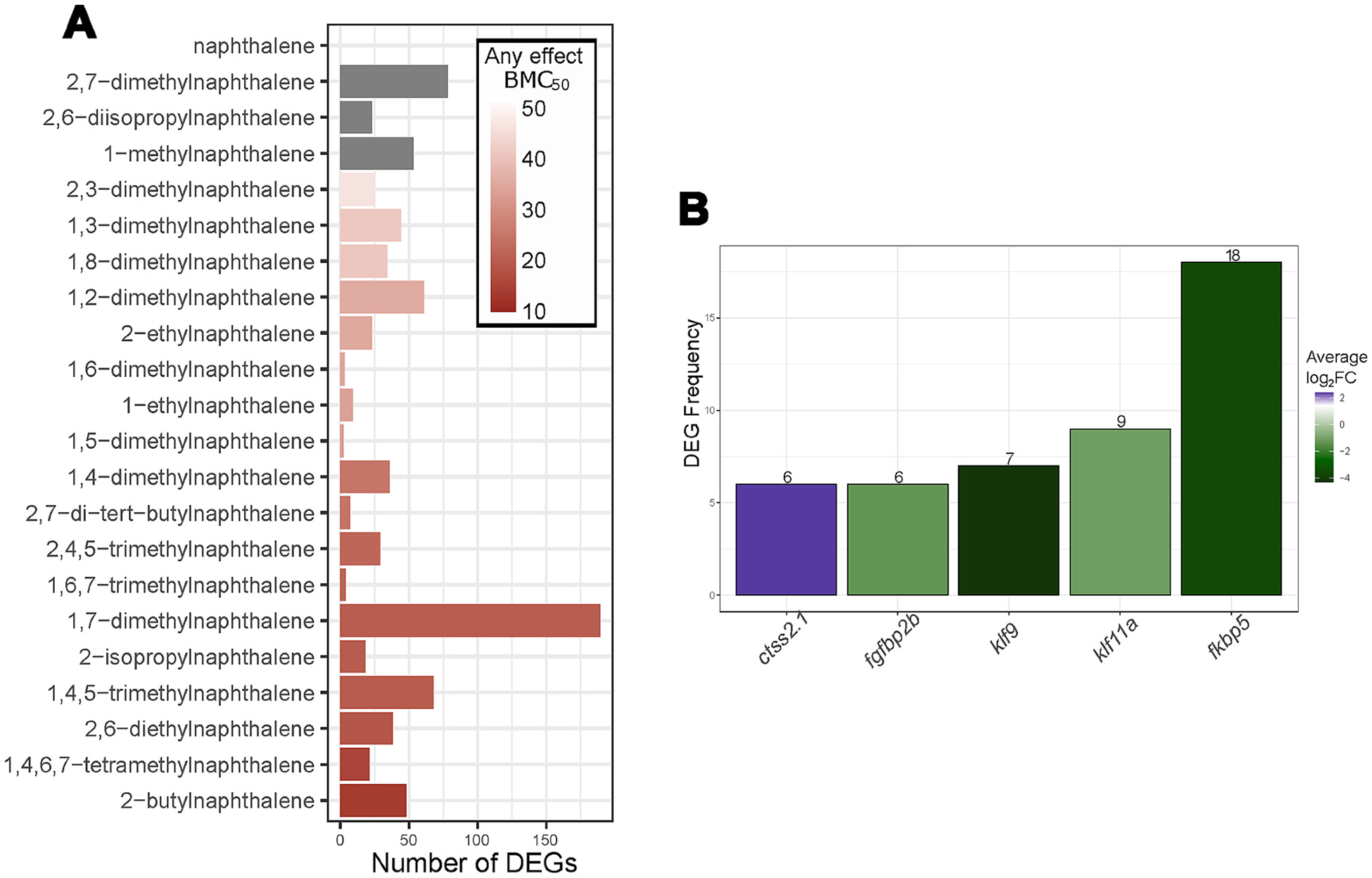
A. Targeted Transciptomics Results. Bar plot showing the number of differentially expressed genes (p-value ≤ 0.05, log_2_ fold change (log_2_FC) ≥ |1|) (DEGs) for each chemical exposure. Color indicates Any effect BMC_50_, grey indicates no calculated BMC_50_. B. Number of times a gene was differentially expressed (p-value ≤ 0.05, log_2_FC ≥ |1|) across all chemical exposures. Bar color represents the average log_2_FC for each gene.

The number of DEGs (p-value ≤ 0.05 and log_2_FC ≥ |1|) identified for each chemical did not correlate with potency as determined by BMC_50_ (Fig. 5A). Low overall DEGs could explain this result. However, the discordance is not unique to this dataset; similar discord was observed for a diverse set of polyfluorinated alkyl substances (Rericha et al., 2024). Notably, 1,7-dimethylnaphthalene had substantially more DEGs than the rest of the chemicals tested even compared to chemicals with a similar BMC_50_ like 1,4,5,7-tetramethylnaphthalene (Fig. 5A). This is especially interesting due to the morphologically distinct effects of 1,7-dimethylnaphthalene, which together may indicate a distinct mode of action for this chemical.

Similarities in the structures of these chemicals suggested that at least some might have the same mode(s) of action. To investigate this, we looked for common DEGs across the chemicals. Five genes were differentially expressed in association with six or more chemicals (*ctss2.1, fgbp2b, klf9, klf11a*, and *fkbp*5) (Fig. 5B). *Fkbp5* was the most frequent DEG, associated with 18 of the 22 chemicals and with an average log_2_FC of −3.57. Additionally, *fkbp5* expression was negatively correlated with increasing potency determined by Spearman’s rank correlation analysis (Table S4). *Fkbp5* expression was strongly correlated with BMC_50_ with a rho of 0.63 and an adjusted p-value of 0.01. *Fkbp5* is part of the FK506 binding protein family and an immunophilin with peptidylprolyl-isomerase activity. It is known to play critical rolls in protein folding, immune regulation and is central to glucocorticoid receptor activity (Eachus et al., 2023; Rein, 2020). Specifically, *fkpb5* is a co-chaperone protein for the glucocorticoid receptor, modulating the affinity of cortisol for this receptor (Schiene-Fischer & Yu, 2001). In zebrafish *fkbp5* is downregulated by fluoxetine and sertraline (selective serotonin reuptake inhibitors), supporting its roll in glucocorticoid receptor activity in zebrafish (Park et al., 2012). Sertraline treatment of zebrafish leads to hypoactivity in the dark phase of a photomotor assay, like that observed for some chemicals in our study (Yang et al., 2021). Additionally, FKBP5 is clinically associated with atrial fibrillation (AF) and Fkbp5 knock out (KO) mice exhibit an increase in AF occurrence (X. Wang et al., 2023). FKBP*5* is a co-chaperone of HSP90′s roll in the degradation of HIF-1a and down regulation of *fkbp5* may stabilize HIF-1a in cardiomyocytes resulting in downstream dysregulation of Ca^2+^ waves.

The next two most frequent DEGs were both in the KLF family (*klf9* and *klf11a*) which are key regulators in embryogenesis and highly conserved between zebrafish and mammals (Nagai et al., 2009; Xue et al., 2015). KLF9 and 11 are members of the same group within the KLF family that interact with the sin3A protein to remodel chromatin and modify histones (Nagai et al., 2009; Xue et al., 2015). Both genes had negative log_2_FCs when differentially expressed and *klf9* had the largest average log_2_FC of the frequent DEGs at −4.3. *Klf11* is downregulated in cancers and KO mice exhibit downregulation of oxidative stress scavengers (Fernandez-Zapico et al., 2003). In zebrafish *klf9* expression fluctuates diurnally and KO was found to increase liver size, decrease oxygen consumption rate (OCR), and upregulate glycolytic genes (Drepanos et al., 2023). Notably, *Klf*9 plays a key role in downstream transcriptomic response from glucocorticoid receptor activity and down regulates the expression of *fkbp5* in zebrafish (Gans et al., 2021).

Both *fgfbp2b* and *ctss2.1* were differentially expressed by six of the chemicals. *Fgfbp2b* encodes for one of five zebrafish fibroblast growth factor binding proteins critical for growth factor signaling during embryogenesis and development (Li et al., 2018). *Ctss2.1* encodes a lysosomal cysteine protein important in immune function and removal of damaged proteins under oxidative stress (Fu et al., 2020; Lalmanach et al., 2020; Lu et al., 2024).

Investigation of frequent DEGs revealed commonly shared transcriptomic responses across many of the alkylated naphthalenes. Correlation between potency and fold change of expression of *fkbp5* suggested a relationship between expression of this gene and alkyl substituted naphthalene toxicity. The key involvement of *klf9* and *fkbp5* in glucocorticoid receptor activity suggested disruption of glucocorticoid signaling by alkyl naphthalene exposure.

### 3.5. Molecular docking

Zebrafish phenotypes and transcriptomics provide important evidence for understanding how alkyl naphthalenes affect development but are insufficient evidence to generate molecular initiation event hypotheses. To this end, we used a reverse molecular docking screen of our alkyl naphthalene chemical structures against a large library predicted human protein binding pockets (Sim et al., 2023). Given that alkyl-substituted naphthalenes may undergo metabolic activation molecular docking of their metabolites would be highly informative. While the measurement or prediction of the metabolites for the 24 chemicals included was outside the scope of this study, this would be a valuable future direction for this work. Using binding pockets predicted from human sequences versus zebrafish may limit interpretation but offsets the considerable uncertainty in structural predictions of zebrafish proteins due to the limited experimentally derived protein structures and the lack of an available database of predicted binding domains for zebrafish protein structures. Given that over 70 % of human protein coding genes have an easily identifiable ortholog in the zebrafish genome, predicted human binding pockets should be reasonably similar to zebrafish binding pocket structures (Howe et al., 2013). Incorporation of human protein binding domains supports our objective to better understand the impact of alkyl naphthalene exposure on human biology and provides a valuable contribution to the field on its own. Follow-up docking was conducted using a zebrafish sequence-derived protein structure prediction to assess whether binding affinity trends were consistent across species for the protein of interest. NT5E docking using experimentally derived human protein structures was also used to validate the results from the predicted structures in the screen.

#### 3.5.1. Reverse molecular docking screen

**Fig. 6.**
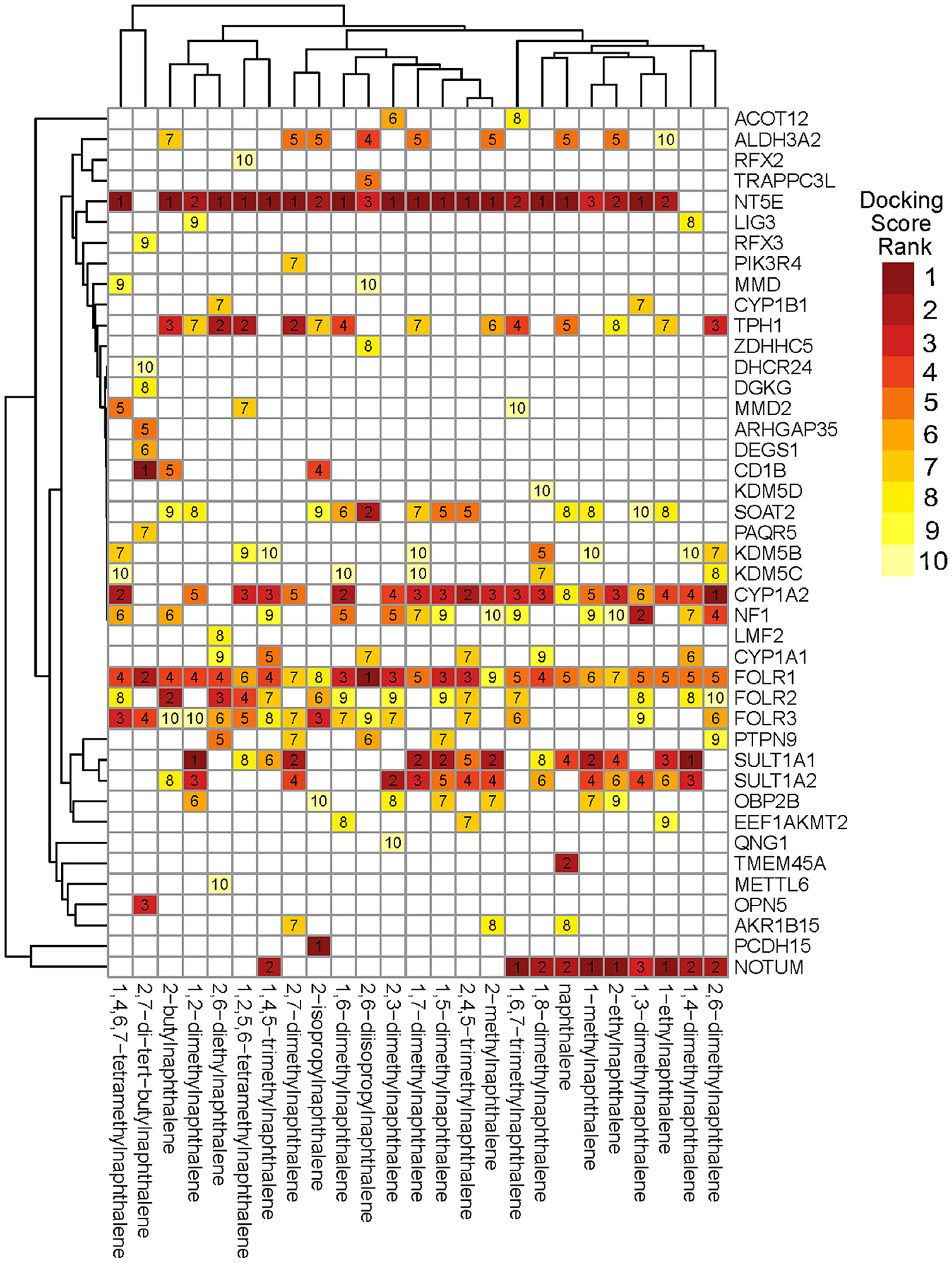
Reserve Molecular Docking Screen Results. Heatmap displaying the rank of predicted binding energies of the 25 naphthalenes. Chemicals were docked against the AlphaFold predicted binding pockets for ~16,000 human proteins. Proteins included in this heatmap ranked among the top 10 for at least one chemical.

Naphthalene and the 24 alkyl-substituted naphthalenes were docked against ~ 30,000 predicted binding pockets from 16,954 AlphaFold predicted protein structures (Sim et al., 2023). The heatmap in Fig. 6 shows trends in binding affinities across chemicals and proteins. The predicted binding affinity ranks were similar across chemicals apart from 2,7-di-*tert*-butylnaphthalene, which was associated with many high-ranking proteins in the top 10 of few or no other chemicals, like OPN5 and CD1B.

Of all the CYP proteins included in the screen, CYP1A2 showed the highest binding affinity for most of the alkyl substituted naphthalenes. Previously, *in vitro* studies found that CYP1A2 had the highest affinity of the CYPs for naphthalene (Cho et al., 2006). Recapitulation of this through reverse molecular docking demonstrated the potential to describe biologically significant interactions. While toxic metabolites from CYP activity are hypothesized to be the cause of naphthalene and monomethylated naphthalene toxicity, previous *in vivo* work demonstrated that naphthalene carcinogenicity was not due to AHR inducible CYPs specifically like CYP1A2 as naphthalenes are poor AHR ligands (Genter et al., 2006). This suggested that although naphthalenes may have a high affinity for binding CYP1A2 may not be induced *in vivo* and thus not contribute to the observed toxicity.

All three Folate acid receptors (FOLR1, FOLR2, and FOLR3) were frequently in the top binding affinities. FOLR1 specifically was in the top 10 predicted binding affinities for all 25 chemicals. Folate receptors are primarily responsible for the transport of folate (Vitamin B) into cells. Folate is essential for cell growth and normal development. In humans, FOLR1 is the main folate receptor associated with this activity. In zebrafish there is one folate receptor (Folr) which is most similar in structure and activity to the human FOLR1 protein (Jones et al., 2017). The folate pathway is tightly regulated during development and its disruption is linked to numerous developmental defects in zebrafish and humans (M. S. Lee et al., 2012; Rosenquist & Finnell, 2001). If alkyl-substituted naphthalenes can bind Folr *in vivo*, it could inhibit cross membrane folate transport and be a potential toxic mechanism that warrants further investigation.

NT5E was among the top three predicted binding affinities for 22 of the chemicals and was predicted to have the highest binding affinity for 15. NT5E encodes protein CD73, an extracellular membrane dimerized protein that converts extracellular AMP to adenosine (Jackson, 2011; Minor et al., 2019). NT5E has been implicated in heart failure and as an immune suppressor with a role in cancer progression (Kordaß et al., 2018; H. Wang et al., 2024). Additionally, CD73 plays an important role in the cAMP pathway linked to the regulation of glucocorticoid receptor signaling through extracellular signal-regulated kinase (ERK) (Jackson, 2011; Kang et al., 2023). CD73 has a zebrafish ortholog by the same name, expressed at 24 hpf in developing zebrafish embryos, making it plausible that these chemicals may interact with it during the exposure window (Thisse & Thisse, 2004). We saw *nt5e* expression, at low read counts, in many of the chemical exposures in our targeted transcriptomics, however, it was detected in only one control sample (SI-T11). While *nt5e* was not significantly differentially expressed in these exposures, likely due to the low read count, we did see decreased expression of another 5′-nucleotidase, *nt5dc2*, in four chemical exposures (1,7 and 2,7-dimethylnaphthalene, 2-ethylnaphthalene, and 1,4,5-trimethylnaphthalene). NT5DC2 is a less well described protein than NT5E, but it also contains a 5′-nucleotidase sequence and is suspected to be involved in adenosine production and/or degradation (Cros-Perrial & Jordheim, 2025). This suggested a potential for disruption of purinergic signaling by our exposures. This, as well as the predicted high affinity for the NT5E protein *in silico*, supported the binding of *nt5e* and disruption of purinergic signaling by alkyl-substituted naphthalenes as a potential mechanism of the observed glucocorticoid related transcriptomic response.

#### 3.5.2. Follow-up molecular docking

**Fig. 7.**
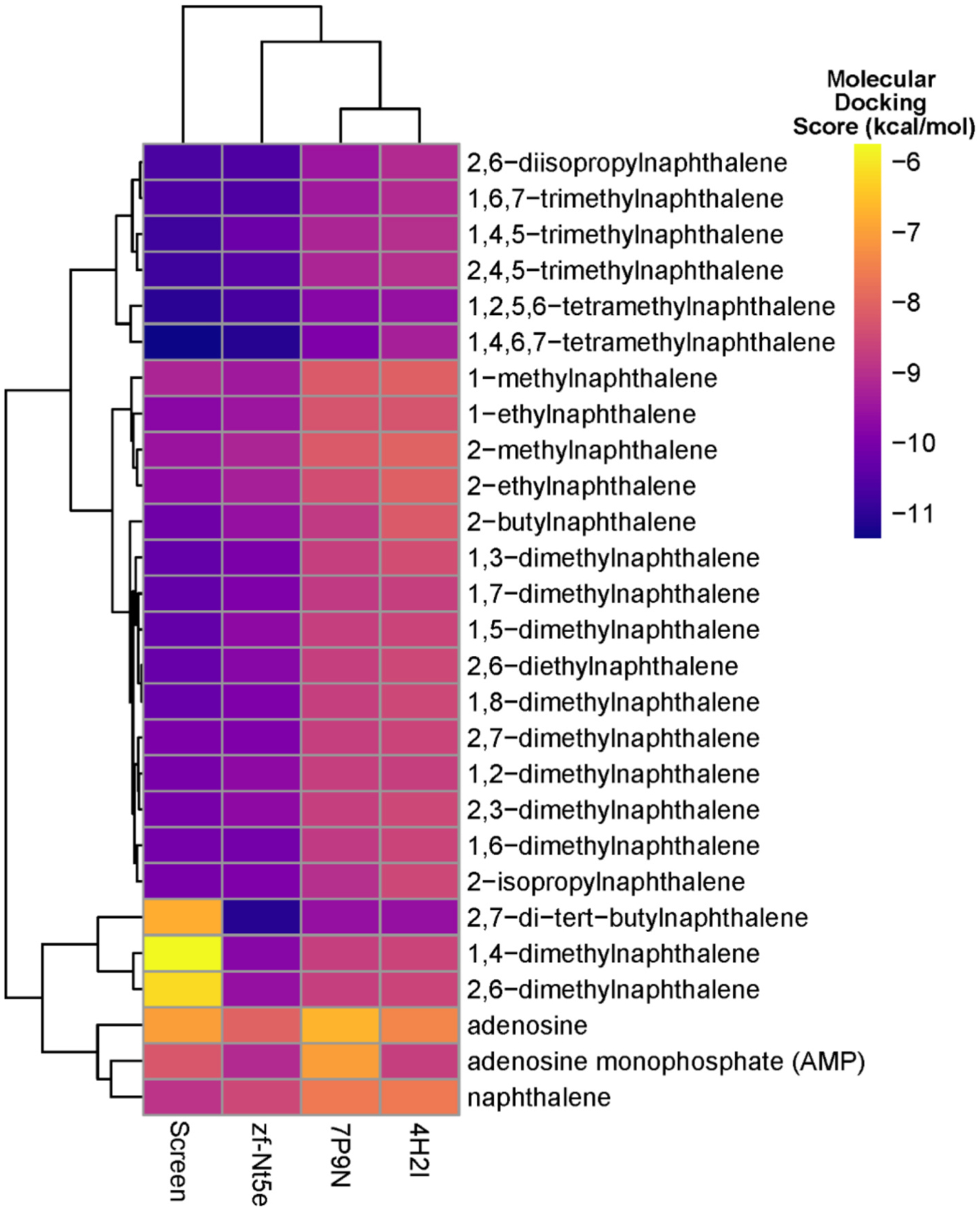
Follow-up Molecular Docking Results. Heatmap displaying the molecular docking scores (kcal/mol) of the 25 naphthalenes docked on the four structures of NT5E. Predicted human protein binding pocket used included in reserve molecular docking screen (Screen), predicted zebrafish protein (zf-Nt5e), open unbound crystal structure (7P9N), and closed conformation bound to adenosine monophosphate (AMP).

Based on our findings from the reverse molecular docking screen using predicted human protein binding sites, we conducted follow-up docking studies on alkyl-substituted naphthalenes and NT5E. All chemicals were docked against the predicted structure for NT5E based on the zebrafish sequence (zf-Nt5e) as well as previously published crystal structures for the open and closed forms of the NT5E (7P9N and 4H2I respectively). Zf-Nt5e was determined to be in the closed conformation based on overlay with both crystal structures. The predicted binding pocket was similarly located to the binding pocket for the closed conformation crystal structure 4H2I. Adenosine and AMP were docked to both the screening binding pocket and follow-up structures to compare the predicted binding affinities of the alkyl-substituted naphthalenes to those of the endogenous ligands. Results from this follow-up docking are shown in Fig. 7.

The follow-up docking scores for all structures recapitulated the trends seen in the screening data across chemicals, with a few notable exceptions; 2,7-ditertbutylnaphthalene, 1,4-dimethylnaphthalene, and 2,6-dimethylnaphthalene. All three of these chemicals had much lower binding affinities in the screening than they did in the follow-up docking. Most of the chemicals tested had higher binding affinities for all NT5E structures than for adenosine and AMP. This was more pronounced in the open conformation human crystal structure 7P9N, where the endogenous ligands had lower binding affinities. The two AlphaFold predicted structures had higher binding affinities across all chemicals, including the endogenous ligands, when compared to the experimentally derived structures, apart from the three chemicals previously mentioned. These results further confirm the findings from the reverse molecular docking screen which suggested that NT5E was a target for alkyl-substituted naphthalenes that could outcompete its endogenous ligand. Collectively, while these findings suggest that alkyl-substituted naphthalenes have a strong affinity for NT5E, additional enzymatic and genetic experiments are required to confirm its involvement in the toxicity of these chemicals in zebrafish and human model systems.

## 4. Conclusions

The vial exposure method developed in this study captured morphological and behavioral effects from developmental exposure to alkyl-substituted naphthalenes by overcoming volatility losses inherent in with 96-well plates. The improved exposure protocol enables more accurate hazard assessment of semi-volatile contaminants, with implications for improved risk assessment. Our results showed high variability in activity across structurally similar compounds and 1,7-dimethylnaphthalene as having distinct morphological effects. A primary hypothesis for naphthalene toxicity involves oxidative damage induced by toxic metabolites requiring aromatic ring oxidation products. Contrary to a previous hypothesis that increased alkyl substitution would lead to decreased toxicity due to decreased ring oxidation, naphthalene and its monomethylated derivatives were not overtly toxic while most of the more highly alkyl substituted derivatives were. Metabolite analysis could confirm the extent of *in vivo* alkyl substituted naphthalene ring oxidation, while measurement of oxidative stress would further clarify the role of this mechanism in the observed toxicity. Conducting these experiments across time, concentration, and structural variants would provide the most insight. This approach would enable the identification of temporal trends in metabolite formation and stress responses, link morphological effects to metabolites and oxidative stress, and reveal the structural features that drive metabolite formation. Variable potency of the alkyl substituted naphthalenes was not explained by logK_ow_ predictions or body burden concentrations, suggesting a non-narcotic mode of action.

Despite low variability in the transcriptomic responses across chemicals, targeted transcriptomics suggested a shared mode of action by many of the chemicals involving glucocorticoid signaling. Measuring cortisol concentrations across time, concentration, and chemical potency could confirm these impacts to glucocorticoid signaling. Reverse molecular docking identified potential protein targets and molecular initiating events. Predicted high-affinity interactions with proteins such as CYP1A2, NT5E, and FOLR1 suggested possible mechanisms underlying the transcriptomic and phenotypic outcomes. Recapitulation of binding affinity trends for NT5E in follow-up docking further supported the hypothesis that alkyl substituted naphthalenes act through disruption of purinergic signaling. Future work will focus on validating the predicted interaction between alkyl substituted naphthalenes and NT5E using a cross-species approach. High-throughput approaches will be used to directly evaluate the binding affinity and functional effects of each alkyl-substituted naphthalene on human NT5E and zebrafish Nt5e (Freundlieb et al., 2014; Senger et al., 2004), and complementary genetic experiments in zebrafish will definitively determine the role of Nt5e in mediating the toxicity of these compounds.

## Supplementary Material

1

2

## Data Availability

The targeted transcriptomic data discussed in this publication have been deposited in NCBI’s Gene Expression Omnibus and are accessible through GEO Series accession number GSE296410

## Appendix A. Supplementary data

Supplementary data to this article can be found online at https://doi.org/10.1016/j.envint.2025.109837.

## References

[R1] Anderson KA, Szelewski MJ, Wilson G, Quimby BD, Hoffman PD, 2015. Modified ion source triple quadrupole mass spectrometer gas chromatograph for polycyclic aromatic hydrocarbon analyses. J. Chromatogr. A 1419, 89–98. 26454790 10.1016/j.chroma.2015.09.054 PMC4721517

[R2] Balik-Meisner MR, Mav D, Phadke DP, Everett LJ, Shah RR, Tal T, Shepard PJ, Merrick BA, Paules RS, 2019. Development of a zebrafish s1500+ sentinel gene set for high-throughput transcriptomics. Zebrafish 16 (4), 331–347. 10.1089/zeb.2018.1720. 31188086 PMC6685209

[R3] Bates D, Mächler M, Bolker B, Walker S, 2015. Fitting linear mixed-effects models using lme4. J. Stat. Softw 67, 1–48. 10.18637/jss.v067.i01.

[R4] Bedell VM, Dubey P, Lee HB, Bailey DS, Anderson JL, Jamieson-Lucy A, Xiao R, Leonard EV, Falk MJ, Pack MA, Mullins M, Farber SA, Eckenhoff RG, Ekker SC, 2025. Zebrafishology, study design guidelines for rigorous and reproducible data using zebrafish. Commun. Biol 8 (1), 739. 10.1038/s42003-025-07496-z. 40360750 PMC12075475

[R5] Bushel PR, Ferguson SS, Ramaiahgari SC, Paules RS, Auerbach SS, 2020. Comparison of Normalization methods for analysis of TempO-Seq Targeted RNA sequencing data. Front. Genet 11. https://www.frontiersin.org/articles/10.3389/fgene.2020.00594. 10.3389/fgene.2020.00594 PMC7325690 32655620

[R6] Casillas G, Scinicariello F, Shoeb MR, Alman B, Carlson-Lynch H, Heit C, Sierco S, & Citra M, 2025. Toxicological Profile for Naphthalene, 1-Methylnaphthalene, and 2-Methylnaphthalene. https://www.atsdr.cdc.gov/toxprofiles/tp67.pdf.

[R7] Chen H, Sheng L, Gong Z, Ru S, Bian H, 2018. Investigation of the molecular mechanisms of hepatic injury upon naphthalene exposure in zebrafish (Danio rerio). Ecotoxicology 27 (6), 650–660. 10.1007/s10646-018-1943-3. 29748829

[R8] Cho TM, Rose RL, Hodgson E, 2006. In vitro metabolism of naphthalene by human liver microsomal cytochrome P450 enzymes. Drug Metab. Dispos 34 (1), 176–183. 10.1124/dmd.105.005785. 16243959

[R9] Cros-Perrial E, Jordheim LP, 2025. A phenotypic journey into NT5DC proteins in cancer and other diseases. Exp. Cell Res 446 (1), 114468. 10.1016/j.yexcr.2025.114468. 39971176

[R10] Di Toro DM, McGrath JA, Hansen DJ, 2000. Technical basis for narcotic chemicals and polycyclic aromatic hydrocarbon criteria. I. Water and tissue. Environ. Toxicol. Chem 19 (8), 1951–1970. 10.1002/etc.5620190803.

[R11] Drepanos L, Gans IM, Grendler J, Guitar S, Fuqua JH, Maki NJ, Tilden AR, Graber JH, Coffman JA, 2023. Loss of Krüppel-like factor 9 deregulates both physiological gene expression and development. Sci. Rep 13 (1), 12239. 10.1038/s41598-023-39453-3. 37507475 PMC10382561

[R12] Eachus H, Oberski L, Paveley J, Bacila I, Ashton J-P, Esposito U, Seifuddin F, Pirooznia M, Elhaik E, Placzek M, Krone NP, Cunliffe VT, 2023. Glucocorticoid receptor regulates protein chaperone, circadian clock and affective disorder genes in the zebrafish brain. Dis. Model. Mech 16 (9), dmm050141. 10.1242/dmm.050141. 37525888 PMC10565112

[R13] Edgar R, Domrachev M, Lash AE, 2002. Gene Expression omnibus: NCBI gene expression and hybridization array data repository. Nucleic Acids Res. 30 (1), 207–210. 10.1093/nar/30.1.207. 11752295 PMC99122

[R14] Everett LJ, Mav D, Phadke DP, Balik-Meisner MR, Shah RR, 2022. Impact of aligner, normalization method, and sequencing depth on TempO-seq accuracy. Bioinf. Biol. Insights 16, 11779322221095216. 10.1177/11779322221095216. PMC9067045 35515009

[R15] Fernandez-Zapico ME, Mladek A, Ellenrieder V, Folch-Puy E, Miller L, Urrutia R, 2003. An mSin3A interaction domain links the transcriptional activity of KLF11 with its role in growth regulation. EMBO J. 22 (18), 4748–4758. 10.1093/emboj/cdg470. 12970187 PMC212736

[R16] Freundlieb M, Zimmermann H, Müller CE, 2014. A new, sensitive ecto-5′-nucleotidase assay for compound screening. Anal. Biochem 446, 53–58. 10.1016/j.ab.2013.10.012. 24144488

[R17] Fu Q, Zhao S, Yang N, Tian M, Cai X, Zhang L, Hu J, Cao M, Xue T, Li C, 2020. Genome-wide identification, expression signature and immune functional analysis of two cathepsin S (CTSS) genes in turbot (Scophthalmus maximus L.). Fish Shellfish Immunol. 102, 243–256. 10.1016/j.fsi.2020.04.028. 32315741

[R18] Gans IM, Grendler J, Babich R, Jayasundara N, Coffman JA, 2021. Glucocorticoid-Responsive Transcription factor Krüppel-like factor 9 Regulates fkbp5 and Metabolism. Front. Cell Dev. Biol 9. 10.3389/fcell.2021.727037. PMC8526736 34692682

[R19] Geier MC, Chlebowski AC, Truong L, Massey Simonich SL, Anderson KA, Tanguay RL, 2018a. Comparative developmental toxicity of a comprehensive suite of polycyclic aromatic hydrocarbons. Arch. Toxicol 92 (2), 571–586. 10.1007/s00204-017-2068-9. 29094189 PMC5820187

[R20] Geier MC, James Minick D, Truong L, Tilton S, Pande P, Anderson KA, Teeguardan J, Tanguay RL, 2018b. Systematic developmental neurotoxicity assessment of a representative PAH Superfund mixture using zebrafish. Toxicol. Appl. Pharmacol 354, 115–125. 10.1016/j.taap.2018.03.029. 29630969 PMC6087484

[R21] Genter MB, Marlowe J, Kevin Kerzee J, Dragin N, Puga A, Dalton TP, Nebert DW, 2006. Naphthalene toxicity in mice and aryl hydrocarbon receptor-mediated CYPs. Biochem. Biophys. Res. Commun 348 (1), 120–123. 10.1016/j.bbrc.2006.07.025. 16876762

[R22] Golzadeh N, Barst BD, Baker JM, Auger JC, McKinney MA, 2021. Alkylated polycyclic aromatic hydrocarbons are the largest contributor to polycyclic aromatic compound concentrations in traditional foods of the Bigstone Cree Nation in Alberta. Canada. Environmental Pollution 275, 116625. 10.1016/j.envpol.2021.116625. 33582641

[R23] Höke H, Zellerhoff R, 1998. Metabolism and toxicity of diisopropylnaphthalene as compared to naphthalene and monoalkyl naphthalenes: a minireview. Toxicology 126 (1), 1–7. 10.1016/S0300-483X(97)00187-X. 9585087

[R24] Honda T, Fukada A, Kiyozumi M, Kojima S, 1987. Identification and determination of urinary metabolites of 2-isopropylnaphthalene in rabbits. Eur. J. Drug Metab. Pharmacokinet 12 (1), 11–16. 10.1007/BF03189856. 3609068

[R25] Honda T, 浄住護雄, 児島昭次, 1990. Alkylnaphthalene. XI.: Pulmonary Toxicity of Naphthalene, 2-Methylnaphthalene, and Isopropylnaphthalenes in mice. Chem. Pharm. Bull 38 (11), 3130–3135. 10.1248/cpb.38.3130. 2085898

[R26] Howe K, Clark MD, Torroja CF, Torrance J, Berthelot C, Muffato M, Collins JE, Humphray S, McLaren K, Matthews L, McLaren S, Sealy I, Caccamo M, Churcher C, Scott C, Barrett JC, Koch R, Rauch G-J, White S, Stemple DL, 2013. The zebrafish reference genome sequence and its relationship to the human genome. Nature 496 (7446), 498–503. 10.1038/nature12111. 23594743 PMC3703927

[R27] Howsam M, Jones KC, 1998. In: Sources of PAHs in the Environment. Springer, Berlin, Heidelberg, pp. 137–174, 10.1007/978-3-540-49697-7_4.

[R28] Hoyberghs J, Bars C, Ayuso M, Van Ginneken C, Foubert K, Van Cruchten S, 2021. DMSO Concentrations up to 1% are safe to be used in the zebrafish embryo developmental toxicity assay. Front. Toxicol 3. 10.3389/ftox.2021.804033. PMC8915880 35295145

[R29] Hudson-Hanley B, Smit E, Branscum A, Hystad P, & Kile ML (2021). Trends in urinary metabolites of polycyclic aromatic hydrocarbons (PAHs) in the non-smoking U.S. population, NHANES 2001–2014. Chemosphere, 276, 130211. doi: 10.1016/j.chemosphere.2021.130211. 33743418 PMC8172479

[R30] Jackson EK, 2011. The 2′,3′-cAMP-adenosine pathway. American Journal of Physiology-Renal Physiology 301 (6), F1160–F1167. 10.1152/ajprenal.00450.2011. 21937608 PMC3233866

[R31] Jochum TK, Stegmüller S, Richling E, 2024. Substance depletion of volatile monoterpenes – a confounding factor for toxicity testing in the Ames fluctuation test. Toxicology 509, 153993. 10.1016/j.tox.2024.153993. 39537009

[R32] Jones RN, Erhard SA, Malham MR, Gen AY, Sullivan K, Olsen KW, Dale RM, 2017. Expression and characterization of the zebrafish orthologue of the human FOLR1 gene during embryogenesis. Gene Expr. Patterns 25–26, 159–166. 10.1016/j.gep.2017.08.002. 28826993

[R33] Kang W, Choi D, Roh J, Jung Y, Ha Y, Yang S, Park T, 2023. The role of cyclic adenosine monophosphate (cAMP) in modulating glucocorticoid receptor signaling and its implications on glucocorticoid-related collagen loss. Int. J. Mol. Sci 24 (12), 12. 10.3390/ijms241210180. PMC10299417 37373328

[R34] Keith LH, 2015. The source of U.S. EPA’s sixteen PAH priority pollutants. Polycycl. Aromat. Compd 35 (2–4), 147–160. 10.1080/10406638.2014.892886.

[R35] Kimmel CB, Ballard WW, Kimmel SR, Ullmann B, Schilling TF, 1995. Stages of embryonic development of the zebrafish. Dev. Dyn 203 (3), 253–310. 10.1002/aja.1002030302. 8589427

[R36] Klüver N, Bittermann K, Escher BI, 2019. QSAR for baseline toxicity and classification of specific modes of action of ionizable organic chemicals in the zebrafish embryo toxicity test. Aquat. Toxicol 207, 110–119. 10.1016/j.aquatox.2018.12.003. 30557756

[R37] Klüver N, Vogs C, Altenburger R, Escher BI, Scholz S, 2016. Development of a general baseline toxicity QSAR model for the fish embryo acute toxicity test. Chemosphere 164, 164–173. 10.1016/j.chemosphere.2016.08.079. 27588575

[R38] Kojima S, Honda T, Nakagawa M, Kiyozumi M, Takadate A, 1982. Urinary metabolites of 2,6-diisopropylnaphthalene in rats. Drug Metab. Dispos 10 (4), 429–433. 6126346

[R39] Kong AX, Johnson M, Eno AF, Pham K, Zhang P, & Geng Y (2024). Proteome-wide reverse molecular docking reveals folic acid receptor as a mediator of PFAS-induced neurodevelopmental toxicity (p. 2024.11.11.623082). bioRxiv. doi: 10.1101/2024.11.11.623082. PMC13099155 41990651

[R40] Kordaß T, Osen W, Eichmüller SB, 2018. Controlling the immune suppressor: transcription factors and MicroRNAs regulating CD73/NT5E. Front. Immunol 9, 813. 10.3389/fimmu.2018.00813. 29720980 PMC5915482

[R41] Lalmanach G, Saidi A, Bigot P, Chazeirat T, Lecaille F, Wartenberg M, 2020. Regulation of the proteolytic activity of cysteine cathepsins by oxidants. Int. J. Mol. Sci 21 (6), 1944. 10.3390/ijms21061944. 32178437 PMC7139492

[R42] Lambert FN, Vivian DN, Raimondo S, Tebes-Stevens CT, Barron MG, 2022. Relationships between aquatic toxicity, chemical hydrophobicity, and mode of action: log kow revisited. Arch. Environ. Contam. Toxicol 83 (4), 326–338. 10.1007/s00244-022-00944-5. 35864329 PMC11375592

[R43] Lee H, Stead JDH, Williams A, Cortés Ramírez SA, Atlas E, Mennigen JA, O’Brien JM, Yauk C, 2024. Empirical characterization of false discovery rates of differentially expressed genes and transcriptomic benchmark concentrations in zebrafish embryos. Environ. Sci. Technol 58 (14), 6128–6137. 10.1021/acs.est.3c10543. 38530926 PMC11008580

[R44] Lee MS, Bonner JR, Bernard DJ, Sanchez EL, Sause ET, Prentice RR, Burgess SM, Brody LC, 2012. Disruption of the folate pathway in zebrafish causes developmental defects. BMC Dev. Biol 12 (1), 1. 10.1186/1471-213X-12-12. 22480165 PMC3410756

[R45] Li Y, Sun S, Ding Z, Yang C, Zhang G, Jiang Q, Zou Y, 2018. Temporal and spatial expression of *fgfbp* genes in zebrafish. Gene 659, 128–136. 10.1016/j.gene.2018.03.032. 29551495

[R46] Lin CY, Wheelock ÅM, Morin D, Baldwin RM, Lee MG, Taff A, Plopper C, Buckpitt A, Rohde A, 2009. Toxicity and metabolism of methylnaphthalenes: Comparison with naphthalene and 1-nitronaphthalene. Toxicology 260 (1), 16–27. 10.1016/j.tox.2009.03.002. 19464565 PMC2687406

[R47] Lu X, Zhang L, Lin G-M, Lu J-G, Cui Z-B, 2024. Analysis of differential gene expression under acute lead or mercury exposure in larval zebrafish using RNA-Seq. Animals 14 (19), 2877. 10.3390/ani14192877. 39409826 PMC11475140

[R48] Minor M, Alcedo KP, Battaglia RA, Snider NT, 2019. Cell type- and tissue-specific functions of ecto-5′-nucleotidase (CD73). Am. J. Phys. Cell Phys 317 (6), C1079–C1092. 10.1152/ajpcell.00285.2019. PMC6957383 31461341

[R49] Moradi M, Hung H, Li J, Park R, Shin C, Alexandrou N, Iqbal MA, Takhar M, Chan A, Brook JR, 2022. Assessment of alkylated and unsubstituted polycyclic aromatic hydrocarbons in air in urban and semi-urban areas in Toronto. Canada. Environmental Science & Technology 56 (5), 2959–2967. 10.1021/acs.est.1c04299. 35148085

[R50] Moran IL, Tidwell L, Barton M, Kile M, Miller P, Rohlman D, Seguinot-Medina S, Ungwiluk B, Waghiyi V, Anderson K, 2023. Diffusive fluxes of persistent organic pollutants between Arctic atmosphere, surface waters and sediments. Sci. Total Environ 892, 164566. 10.1016/j.scitotenv.2023.164566. 37270011 PMC10330832

[R51] Morshead ML, Truong L, Simonich MT, Moran JE, Anderson KA, Tanguay RL, 2025. Developmental toxicity of alkylated PAHs and substituted phenanthrenes: Structural nuances drive diverse toxicity and AHR activation. Chemosphere 370, 143894. 10.1016/j.chemosphere.2024.143894. 39643011 PMC11732715

[R52] Nagai R, Friedman S, Kasuga M, 2009. The Biology of Krüppel-like Factors. Springer.

[R53] O’Boyle NM, Banck M, James CA, Morley C, Vandermeersch T, Hutchison GR, 2011. Open Babel: an open chemical toolbox. J. Cheminf 3 (1), 33. 10.1186/1758-2946-3-33. PMC3198950 21982300

[R54] Park J-W, Heah TP, Gouffon JS, Henry TB, Sayler GS, 2012. Global gene expression in larval zebrafish (*Danio rerio*) exposed to selective serotonin reuptake inhibitors (fluoxetine and sertraline) reveals unique expression profiles and potential biomarkers of exposure. Environ. Pollut 167, 163–170. 10.1016/j.envpol.2012.03.039. 22575097

[R55] Peng B, Dong Q, Li F, Wang T, Qiu X, Zhu T, 2023. A systematic review of polycyclic aromatic hydrocarbon derivatives: occurrences, levels, biotransformation, exposure biomarkers, and toxicity. Environ. Sci. Technol 57 (41), 15314–15335. 10.1021/acs.est.3c03170. 37703436

[R56] Pettersen EF, Goddard TD, Huang CC, Couch GS, Greenblatt DM, Meng EC, Ferrin TE, 2004. UCSF Chimera—A visualization system for exploratory research and analysis. J. Comput. Chem 25 (13), 1605–1612. 10.1002/jcc.20084. 15264254

[R57] Preuss R, Angerer J, Drexler H, 2003. Naphthalene—An environmental and occupational toxicant. Int. Arch. Occup. Environ. Health 76 (8), 556–576. 10.1007/s00420-003-0458-1. 12920524

[R58] Proença S, Escher BI, Fischer FC, Fisher C, Grégoire S, Hewitt NJ, Nicol B, Paini A, Kramer NI, 2021. Effective exposure of chemicals in *in vitro* cell systems: a review of chemical distribution models. Toxicol. In Vitro 73, 105133. 10.1016/j.tiv.2021.105133. 33662518

[R59] Qiao M, Fu L, Li Z, Liu D, Bai Y, Zhao X, 2020. Distribution and ecological risk of substituted and parent polycyclic aromatic hydrocarbons in surface waters of the Bai, Chao, and Chaobai rivers in northern China. Environ. Pollut 257, 113600. 10.1016/j.envpol.2019.113600. 31748130

[R60] R Core Team. (2023). R: A language and environment for statistical computing [Computer software]. https://www.R-project.org/.

[R61] Rein T, 2020. Peptidylprolylisomerases, protein folders, or scaffolders? The example of FKBP51 and FKBP52. Bioessays 42 (7), 1900250. 10.1002/bies.201900250. 32323357

[R62] Rericha Y, St. Mary L, Truong L, McClure R, Martin JK, Leonard SW, Thunga P, Simonich MT, Waters KM, Field JA, Tanguay RL, 2024. Diverse PFAS produce unique transcriptomic changes linked to developmental toxicity in zebrafish. Front. Toxicol 6, 1425537. 10.3389/ftox.2024.1425537. 39104825 PMC11298493

[R63] Ritchie ME, Phipson B, Wu D, Hu Y, Law CW, Shi W, Smyth GK, 2015. Limma powers differential expression analyses for RNA-sequencing and microarray studies. Nucleic Acids Res. 43 (7), e47. 25605792 10.1093/nar/gkv007 PMC4402510

[R64] Rosenquist TH, Finnell RH, 2001. Genes, folate and homocysteine in embryonic development. Proc. Nutr. Soc 60 (1), 53–61. 10.1017/S0029665101000088. 11310424

[R65] Rude CI, Smith JN, Scott RP, Schultz KJ, Anderson KA, Tanguay RL, 2025. A mixture parameterized biologically based dosimetry model to predict body burdens of polycyclic aromatic hydrocarbons in developmental zebrafish toxicity assays. Toxicol. Sci 205 (2), 326–343. 10.1093/toxsci/kfaf039. 40117221 PMC12118961

[R66] Schiene-Fischer C, Yu C, 2001. Receptor accessory folding helper enzymes: the functional role of peptidyl prolyl *cis*/*trans* isomerases. FEBS Lett. 495 (1), 1–6. 10.1016/S0014-5793(01)02326-2. 11322937

[R67] Schöning-Stierand K, Diedrich K, Ehrt C, Flachsenberg F, Graef J, Sieg J, Penner P, Poppinga M, Ungethüm A, Rarey M, 2022. ProteinsPlus: a comprehensive collection of web-based molecular modeling tools. Nucleic Acids Res. 50 (W1), W611–W615. 10.1093/nar/gkac305. 35489057 PMC9252762

[R68] Senger MR, Rico EP, Dias RD, Bogo MR, Bonan CD, 2004. Ecto-5′-nucleotidase activity in brain membranes of zebrafish (Danio rerio). Comp. Biochem. Physiol. B Biochem. Mol. Biol 139 (2), 203–207. 10.1016/j.cbpc.2004.07.011. 15465666

[R69] Shankar P, Geier MC, Truong L, McClure RS, Pande P, Waters KM, Tanguay RL, 2019. Coupling genome-wide transcriptomics and developmental toxicity profiles in zebrafish to characterize polycyclic aromatic hydrocarbon (PAH) hazard. Int. J. Mol. Sci 20 (10), 2570. 10.3390/ijms20102570. 31130617 PMC6566387

[R70] Sim J, Kwon S, Seok C, 2023. HProteome-BSite: predicted binding sites and ligands in human 3D proteome. Nucleic Acids Res. 51 (D1), D403–D408. 10.1093/nar/gkac873. 36243970 PMC9825455

[R71] Souza MCO, Rocha BA, Cruz JC, Palir N, Campíglia AD, Domingo JL, Barbosa F, 2023. Risk characterization of human exposure to polycyclic aromatic hydrocarbons in vulnerable groups. Sci. Total Environ 892, 164219. 10.1016/j.scitotenv.2023.164219. 37230361

[R72] Stadnicka-Michalak J, Bramaz N, Schönenberger R, Schirmer K, 2021. Predicting exposure concentrations of chemicals with a wide range of volatility and hydrophobicity in different multi-well plate set-ups. Sci. Rep 11 (1), 4680. 10.1038/s41598-021-84109-9. 33633258 PMC7907087

[R73] Thisse B, Thisse C, 2004. Fast Release Clones: a High Throughput Expression Analysis. ZFIN Direct Data Submission. https://zfin.org/ZDB-PUB-040907-1#summary.

[R74] Truong L, Bugel SM, Chlebowski A, Usenko CY, Simonich MT, Simonich SLM, Tanguay RL, 2016. Optimizing multi-dimensional high throughput screening using zebrafish. Reprod. Toxicol 65, 139–147. 10.1016/j.reprotox.2016.05.015. 27453428 PMC5067206

[R75] Truong L, Rericha Y, Thunga P, Marvel S, Wallis D, Simonich MT, Field JA, Cao D, Reif DM, Tanguay RL, 2022. Systematic developmental toxicity assessment of a structurally diverse library of PFAS in zebrafish. J. Hazard. Mater 431, 128615. 10.1016/j.jhazmat.2022.128615. 35263707 PMC8970529

[R76] US Environmental Protection Agency (EPA), 2020. Benchmark Dose Software (BMDS). User Guide Version 3 (2). https://assessments.epa.gov/bmds/document/&deid%3D353980.

[R77] US EPA, 2024. Estimation Programs Interface Suite^™^ for Microsoft^®^ Windows (Version 4.11) [Computer software]. https://www.epa.gov/tsca-screening-tools/download-epi-suitetm-estimation-program-interface-v411.

[R78] Van Winkle LS, Isaac JM, Plopper CG, 1997. Distribution of epidermal growth factor receptor and ligands during bronchiolar epithelial repair from naphthalene-induced Clara cell injury in the mouse. Am. J. Pathol 151 (2), 443–459. 9250157 PMC1857992

[R79] Varadi M, Anyango S, Deshpande M, Nair S, Natassia C, Yordanova G, Yuan D, Stroe O, Wood G, Laydon A, Žídek A, Green T, Tunyasuvunakool K, Petersen S, Jumper J, Clancy E, Green R, Vora A, Lutfi M, Velankar S, 2022. AlphaFold Protein Structure Database: Massively expanding the structural coverage of protein-sequence space with high-accuracy models. Nucleic Acids Res. 50 (D1), D439–D444. 10.1093/nar/gkab1061. 34791371 PMC8728224

[R80] Wang D, Bruyneel B, Kamelia L, Wesseling S, Rietjens IMCM, Boogaard PJ, 2020. In vitro metabolism of naphthalene and its alkylated congeners by human and rat liver microsomes via alkyl side chain or aromatic oxidation. Chem. Biol. Interact 315, 108905. 10.1016/j.cbi.2019.108905. 31765606

[R81] Wang H, Cai P, Yu X, Li S, Zhu W, Liu Y, Wang D, 2024. Bioinformatics identifies key genes and potential drugs for energy metabolism disorders in heart failure with dilated cardiomyopathy. Front. Pharmacol 15. 10.3389/fphar.2024.1367848. PMC10952830 38510644

[R82] Wang X, Song J, Yuan Y, Li L, Abu-Taha I, Heijman J, Sun L, Dobrev S, Kamler M, Xie L, Wehrens XHT, Horrigan FT, Dobrev D, Li N, 2023. Downregulation of FKBP5 Promotes Atrial Arrhythmogenesis. Circ. Res 133 (1), e1–e16. 10.1161/CIRCRESAHA.122.322213. 37154033 PMC10330339

[R83] Warren DL, Brown DL, Buckpitt AR, 1982. Evidence for cytochrome *P*-450 mediated metabolism in the bronchiolar damage by naphthalene. Chem. Biol. Interact 40 (3), 287–303. 10.1016/0009-2797(82)90152-1. 7083396

[R84] Williams AJ, Grulke CM, Edwards J, McEachran AD, Mansouri K, Baker NC, Patlewicz G, Shah I, Wambaugh JF, Judson RS, Richard AM, 2017. The CompTox Chemistry Dashboard: a community data resource for environmental chemistry. J. Cheminf 9 (1), 61. 10.1186/s13321-017-0247-6. PMC5705535 29185060

[R85] Wilson LB, McClure RS, Waters KM, Simonich MT, Tanguay RL, 2022. Concentration-response gene expression analysis in zebrafish reveals phenotypically-anchored transcriptional responses to retene. Front. Toxicol 4. https://www.frontiersin.org/articles/10.3389/ftox.2022.950503. 10.3389/ftox.2022.950503 PMC9453431 36093370

[R86] Wnorowski A, Harnish D, Jiang Y, Celo V, Dabek-Zlotorzynska E, Charland J-P, 2022. Assessment and characterization of alkylated PAHs in selected sites across Canada. Atmos. 13 (8), 8. 10.3390/atmos13081320.

[R87] Xue Y, Gao S, Liu F, 2015. Genome-wide analysis of the zebrafish Klf family identifies two genes important for erythroid maturation. Dev. Biol 403 (2), 115–127. 10.1016/j.ydbio.2015.05.015. 26015096

[R88] Yang H, Liang X, Zhao Y, Gu X, Mao Z, Zeng Q, Chen H, Martyniuk CJ, 2021. Molecular and behavioral responses of zebrafish embryos/larvae after sertraline exposure. Ecotoxicol. Environ. Saf 208, 111700. 10.1016/j.ecoenv.2020.111700. 33396031

[R89] Yu H, Liu Y, Han C, Fang H, Weng J, Shu X, Pan Y, Ma L, 2021. Polycyclic aromatic hydrocarbons in surface waters from the seven main river basins of China: Spatial distribution, source apportionment, and potential risk assessment. Sci. Total Environ 752, 141764. 10.1016/j.scitotenv.2020.141764. 32898799

[R90] Zhang Y, Parmigiani G, Johnson WE, 2020. ComBat-seq: batch effect adjustment for RNA-seq count data. NAR Genomics Bioinf. 2 (3), lqaa078. 10.1093/nargab/lqaa078. PMC7518324 33015620

