# Corrigendum to “Chemical structure drives developmental toxicity of alkyl-substituted naphthalenes in zebrafish” [Environ. Int. 204 (2025) 109837]

**DOI:** 10.1016/j.envint.2026.110354

**Published:** 2026-06-13

**Authors:** Mackenzie L. Morshead, Lisa Truong, Yijie Geng, Steven J. Carrell, Richard Scott, Kim A. Anderson, Robyn L. Tanguay

**Affiliations:** Sinnhuber aquatic Research Center, Department of Environmental and Molecular Toxicology and the Oregon State University Superfund Center, Oregon State University, ALS 1007, Corvallis, OR 97331, USA

The authors regret that in the original article, Yijie Geng was inadvertently omitted from the author list. The author list has now been updated. Yijie Geng significantly contributed to the data generation and analysis, which was not properly credited in the original manuscript. Additionally, the following changes should be made in the text to properly cite Geng lab's publications. In section 2.6, the first two sentences should be revised into the following sentence [Naphthalene and alkyl-substituted naphthalene structures were docked to predicted binding sites of AlphaFold2 predicted structures (Sim et al., 2023; Varadi et al., 2022) as previously reported (Kong et al., 2024)]. In section 3.5, the second sentence should be revised as follows [To this end, we used a reverse molecular docking screen of our alkyl naphthalene chemical structures against a large library predicted human protein binding pockets (Sim et al., 2023) as previously described (PMID: 39605555; PMID: 41990651)]. The authors apologize for this error and state that these changes do not change the scientific conclusions of the article in any way.

