# Supplementary material for: Chemical structure drives developmental toxicity of alkyl-substituted naphthalenes in zebrafish
Source: Environ Int. Author manuscript; Available in PMC 2025 Oct 28. (PMC12560828; doi:10.1016/j.envint.2025.109837)
Supplement: 2 [file NIHMS2119078-supplement-2.docx]

**Supplemental Table S1:** Sources for analytical standards

| 161 PAH Standard Vendors. | |
| --- | --- |
| Vendor | Location |
| Sigma Aldrich | St. Louis, MO |
| Santa Cruz Biotech | Dallas, TX |
| Chiron | Tampa, FL |
| Tokyo Chemical Industry | Portland, OR |
| Accustandard | New Haven, CT |
| MRI Global | Kansas City, MO |
| ThermoFisher | Waltham, MA |
| CDN Isotopes | Pointe-Claire, Quebec |
| Toronto Research Chemicals (now owned by LCG). | Teddlington, Middlesex, UK |

**Supplemental Table S2**: linear model parameters for models shown in Supplemental Figure 2.

| Model A | | | | |
| --- | --- | --- | --- | --- |
|  | Estimate | Upper | Lower | p-value |
| Intercept | 61.7 | 95.7 | 27.6 | 1.45x10^-3^ |
| Slope | -7.07 | 0.103 | -14.2 | 0.0529 |
| Model B | | | | |
| Intercept | 140 | 23.0 | 6.10 | 2.04x10^-5^ |
| Slope | -24.4 | 5.02 | -4.87 | 2.05x10^-4^ |

**Supplemental Table S3**: Detailed instrument conditions for chemical analysis of body burden concentrations.

| **161 PAH Method** | | | |
| --- | --- | --- | --- |
| **Carrier Gas** | | | |
| Helium (99.99%) | | | |
| **Oven Program** | | **Inlet Settings** | |
| hold 60°C for 1 min  ramp 40°C/min to 180°C  ramp 3°C/min to 230°C  ramp 1.5°C to 235°C  ramp 15°C/min to 280°C, hold for 10min  ramp 6°C/min to 298°C  ramp 16°C/min to 350°C, hold 4min  Total run time: 47.25 min | | **Mode** | Pulsed Splitless |
|  |  | **Injection Temperature (°C)** | 320 |
|  |  | **Pulse Pressure** | 35 psi Until 0.3 min |
|  |  | **Purge Flow** | 25 mL/min at 0.7 min |
| **Column Settings & Specifications** | | **MSD Settings** | |
| J&W Select PAH, Part # CP7462, L 30 m, ID 0.25 mm, Film 0.15 µm | | **MSD Transfer Line Temp** | 320°C |
|  |  | **Source Temp** | 340°C |
| **Mode** | Constant Flow | **Collision Gas (purity)** | Nitrogen (99.99%) |
| **Flow Rate** | 2 mL/min | **Flow Rate** | 1.5 mL/min |


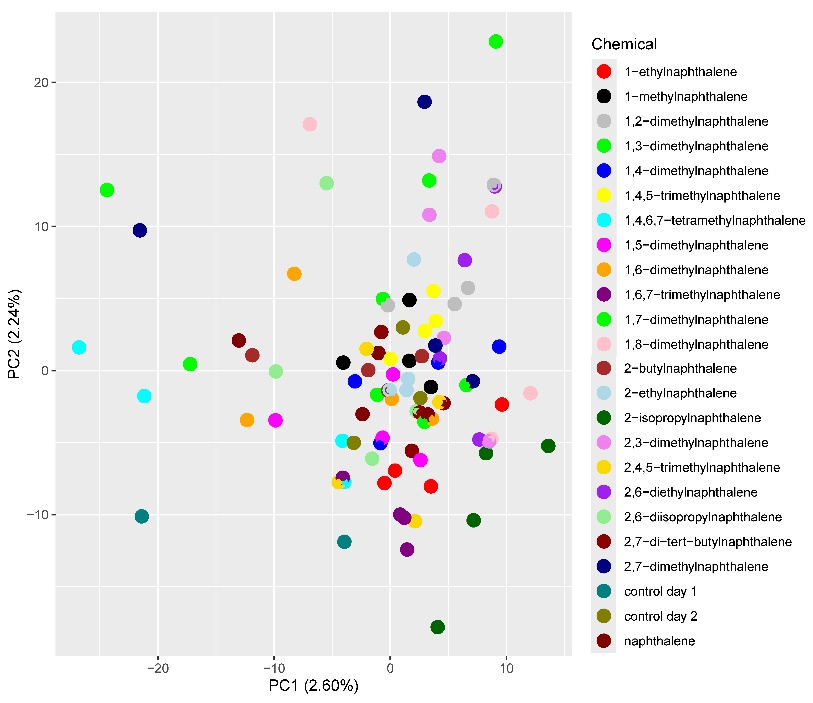


**Supplemental Table S4:** Spearman’s rank correlation assessment for genes differentially expressed in at least 25% of chemical exposures.

| Gene | Rho | p-value | Holm adjusted p-value |
| --- | --- | --- | --- |
| *fkbp5* | 0.6302 | 0.0017 | 0.0083 |
| *klf9* | 0.4751 | 0.0255 | 0.1018 |
| *klf11a* | 0.3488 | 0.1116 | 0.3348 |
| *ctss2.1* | -0.0878 | 0.6977 | 1.0000 |
| *fgfbp2b* | 0.0051 | 0.9820 | 1.0000 |


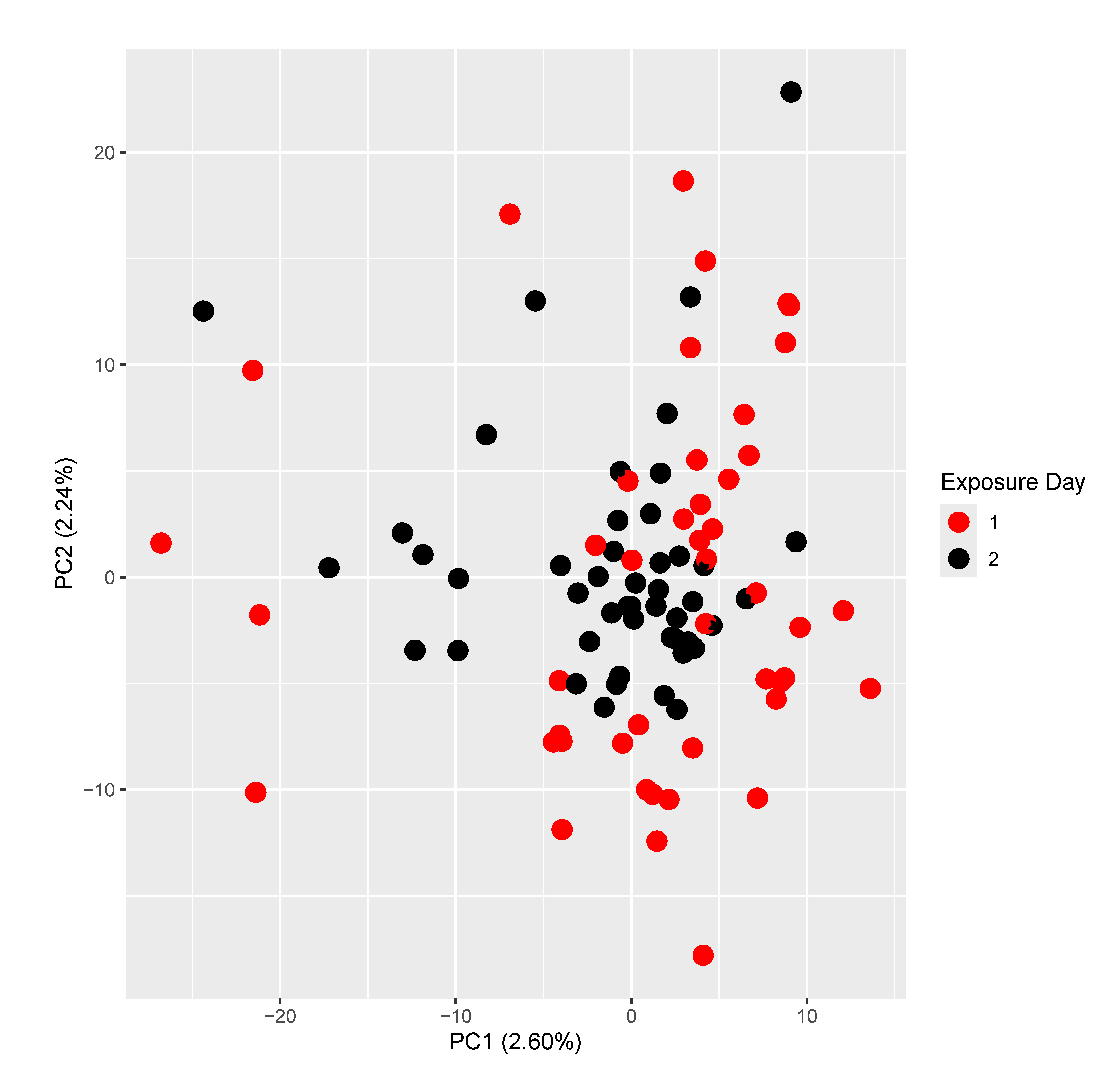


**Supplemental Figure 1:** Principal component analysis (PCA) plot of batch corrected interquartile range (IQR) normalized targeted transcriptomics read counts. Exposure day is indicated by color.


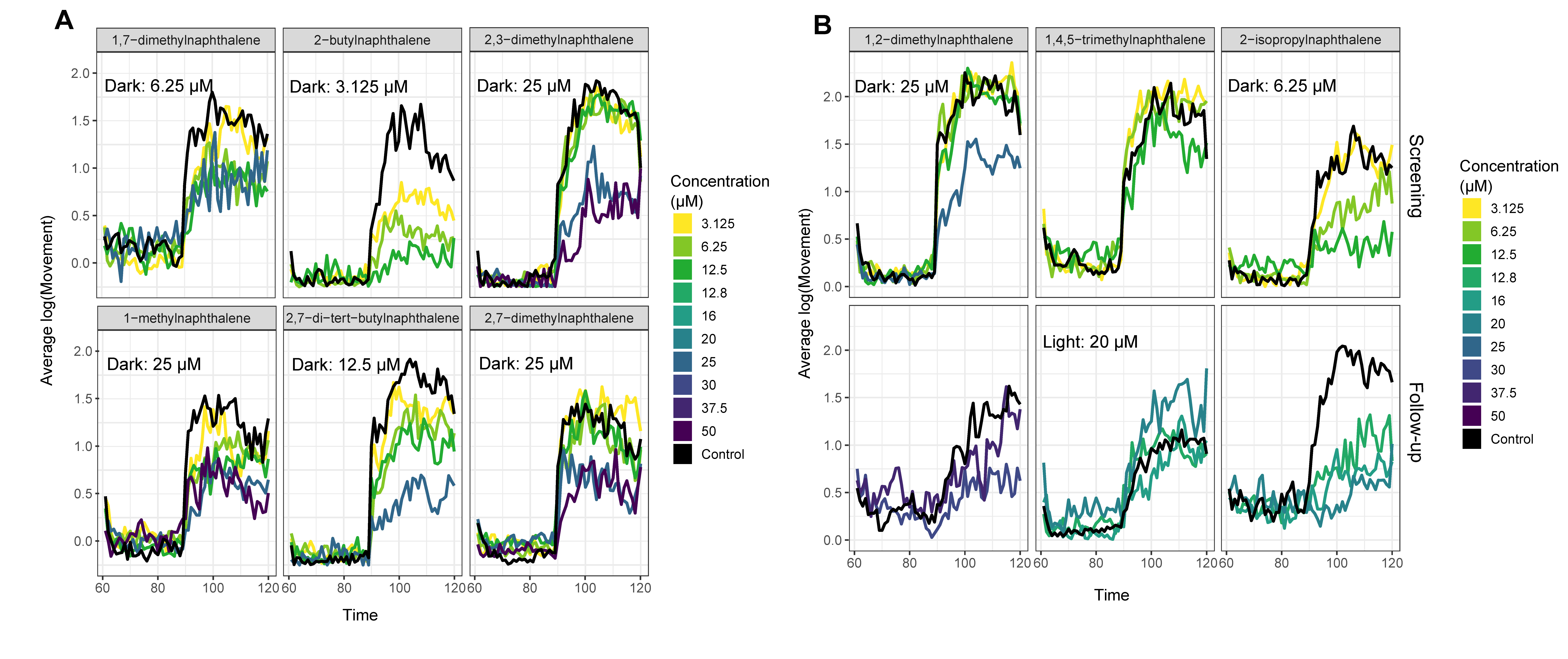
**Supplemental Figure S2:** **A.** Average log(movement) over time for each chemical with a lowest effect level (LEL). LEL values are shown. Chemicals which were did not require additional follow up exposures. **B.** Chemicals which were included in follow-up exposures, traces are shown seperately by day as behavioral response is variable by day.


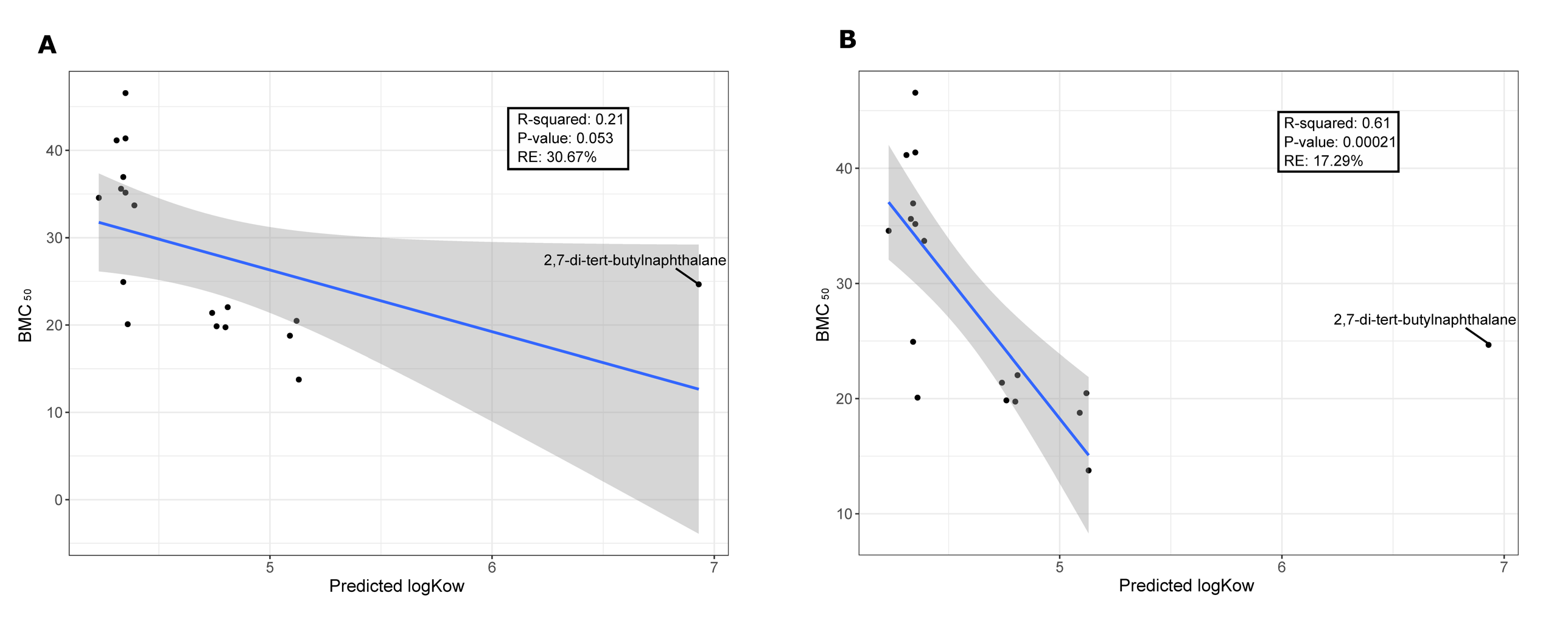
**Supplemental Figure S3:** **A.** Linear model for predicted logKow vs. BMC_50_ for all chemicals with a calculated BMC_50_ value. Grey area represents the 95% confidence interval. **B.** Linear model for predicted logKow vs. BMD_50_ removing the logKow outlier, 2,7-di-tert-butylnaphathalene. Grey area represents the 95% confidence interval.


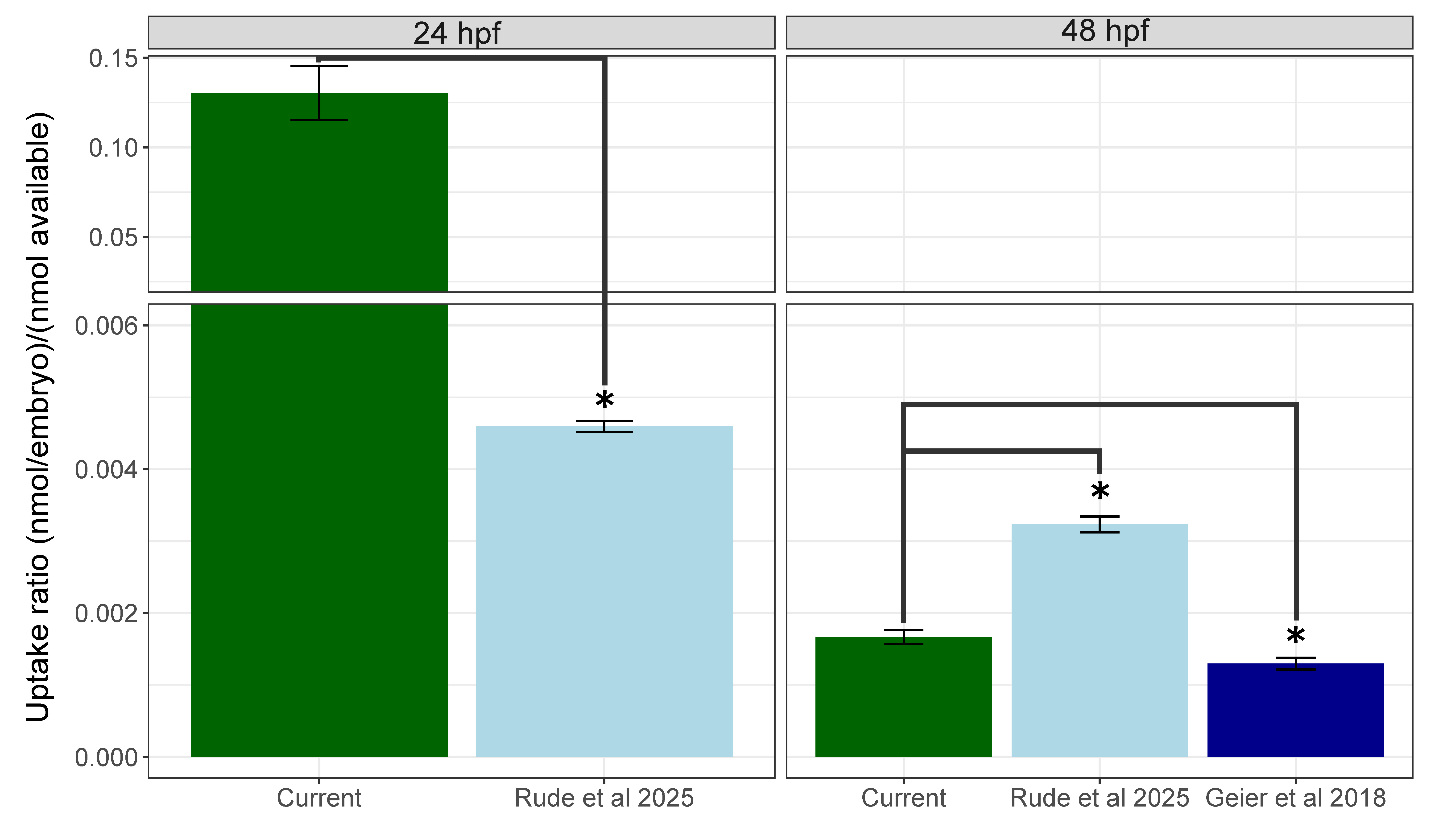


**Supplemental Figure S4:** Bar plot comparing measured body burden concentration uptake rations of naphthalene nmol/embryo per nmol available across the current study, using vial exposures, and Rude et al. 2025 and Geier et al 2018, using 96-well exposures. Error bars indicate one standard deviation. Asterisks indicate p-value ≤0.05.
